# Accommodation of Dental Variations During Jaw Growth in Ungulate Mammals

**DOI:** 10.1002/jez.b.23321

**Published:** 2025-08-05

**Authors:** Helder Gomes Rodrigues, Jules Chabot, Thomas Cucchi, Guillaume Billet

**Affiliations:** ^1^ Centre de Recherche en Paléontologie— Paris (CR2P), UMR CNRS 7207, CP38, Muséum National d'Histoire Naturelle Sorbonne Université Paris France; ^2^ BioArchéologie, Interactions Sociétés Environnements (BioArch), UMR CNRS 7209, Muséum National d'Histoire Naturelle Paris France

**Keywords:** constraints, crown height, eruption, geometric morphometrics, ontogeny, teeth‐palate complex

## Abstract

The growth of teeth and jaw bones is intimately linked in vertebrates, especially in mammals due to their specialized dentition and limited body growth. However, the relative patterns of growth and level of integration (i.e., co‐variation) of these structures are insufficiently known, which hinders our ability to understand how the jaw bones accommodate the diverse dental shapes and eruption patterns observed in mammals. Here, we studied the cranial ontogenetic series of 23 ungulate species among artiodactyls, perissodactyls, and hyracoids having different dental shapes and eruption patterns. We evaluated the variation of the teeth‐palate complex, as well as the co‐variation of teeth and palate during growth using 3D geometric morphometrics. We found a major ontogenetic component common to all the species studied, corresponding to an elongated dental row relative to the palate in juveniles and vice versa in adults. This pattern agrees with the prolonged growth of the palate as compared to teeth during development but is also reminiscent of an intraspecific allometric pattern previously observed in some dwarf ungulates. Moreover, most artiodactyls, especially ruminants, departed from other ungulates in having a higher co‐variation between the dental row and the palate. This stronger integration seen in ruminants might be associated with their inherited rapid growth and relatively fast eruption pattern. This is in contrast to ungulates with late eruption of last molars, whose teeth‐palate complex might be less constrained, but further investigation is needed to substantiate these hypotheses and better understand the factors influencing covariations within the upper jaw.

## Graphical abstract

Using geometric morphometrics, we compared the covariation pattern of the dentition and the palate during growth in 23 ungulate species.

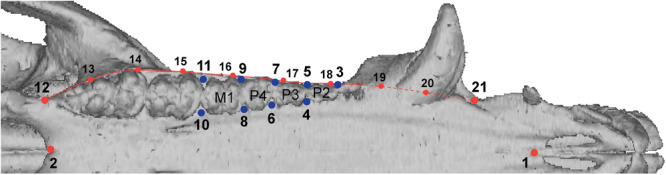

## 1 Introduction

Among vertebrates, mammals stand out with the dual nature of their specialized masticatory apparatus (Luo 2007; Ungar 2010; Berkovitz and Shellis 2018; Martin and Koenigswald 2021). On one hand, the specific nature of mammalian teeth lies in their simple mode of replacement (i.e., only two sets of teeth, the permanent replacing the deciduous ones) and their limited number in the jaw, which are tightly associated to a determinate skull growth (Luo et al. 2004; Kemp 2005). On the other hand, these apparent simplifications are accompanied by a more complex shape of their dentition, which allows mammals to access a wide range of food (Kemp 2005; Luo 2007; Ungar 2010), including the consumption of a huge variety of plants. Specializations for tough and abrasive plants consumption frequently involve a high complexification of cheek teeth shape for efficient food processing, onto which a prolonged to continuous growth of teeth can be superimposed to withstand wear (Janis and Fortelius 1988; von Koenigswald 2011; Renvoisé and Michon 2014; Martin et al. 2021). The tempo of dental eruption and replacement can also be modified to make the dentition either more rapidly efficient or more durable (Janis and Fortelius 1988; Smith 2000; Gomes Rodrigues et al. 2017a; Monson and Hlusko 2018). In the context of a limited cranial growth, it can be asked how the mammalian jaw has accommodated the evolution of dental specializations, including variable crown heights and paces of dental eruption.

Past studies have clearly emphasized the non‐independent developmental relationships between teeth and bone during their growth (Grüneberg 1937; Butler 1956; Lungová et al. 2011; Alfaqeeh et al. 2013; Renvoisé et al. 2017). The size and shape of teeth and alveolar bone are mostly genetically determined (Atchley and Hall 1991; Paradis et al. 2013; Jernvall and Thesleff 2000; Gomes Rodrigues et al. 2013; Lungová et al. 2011), but the growth of the tooth germ is constrained by the surrounding bone, which can affect the size of the tooth (Grüneberg 1937; Alfaqeeh et al. 2013) and to a lesser extent, its shape (Renvoisé et al. 2017). Conversely, dental growth involves intense bone resorption and remodelling, especially during tooth eruption or drift along the jaw (Wise and King 2008; Henneman et al. 2008; Lentle and Hume 2010; Gomes Rodrigues et al. 2012). Previous studies have also shown that the shape and size of the dentition and the jaw seem intimately linked (e.g., Renvoisé et al. 2017; Phen et al. 2018; Billet and Bardin 2021; Sadier et al. 2023). These works demonstrate that a mutual influence exists between teeth and jaw bones, and that these two units represent an intricate “paradox of evo‐devo integration and autonomy”, as expressed in Phen et al. (2018). Yet, we still have a limited understanding of their coordinated growth and their integration (i.e., covariation) since these aspects remain poorly explored quantitatively, especially across key moments of the jaw setting, such as dental eruption and replacement.

**Figure 1:**
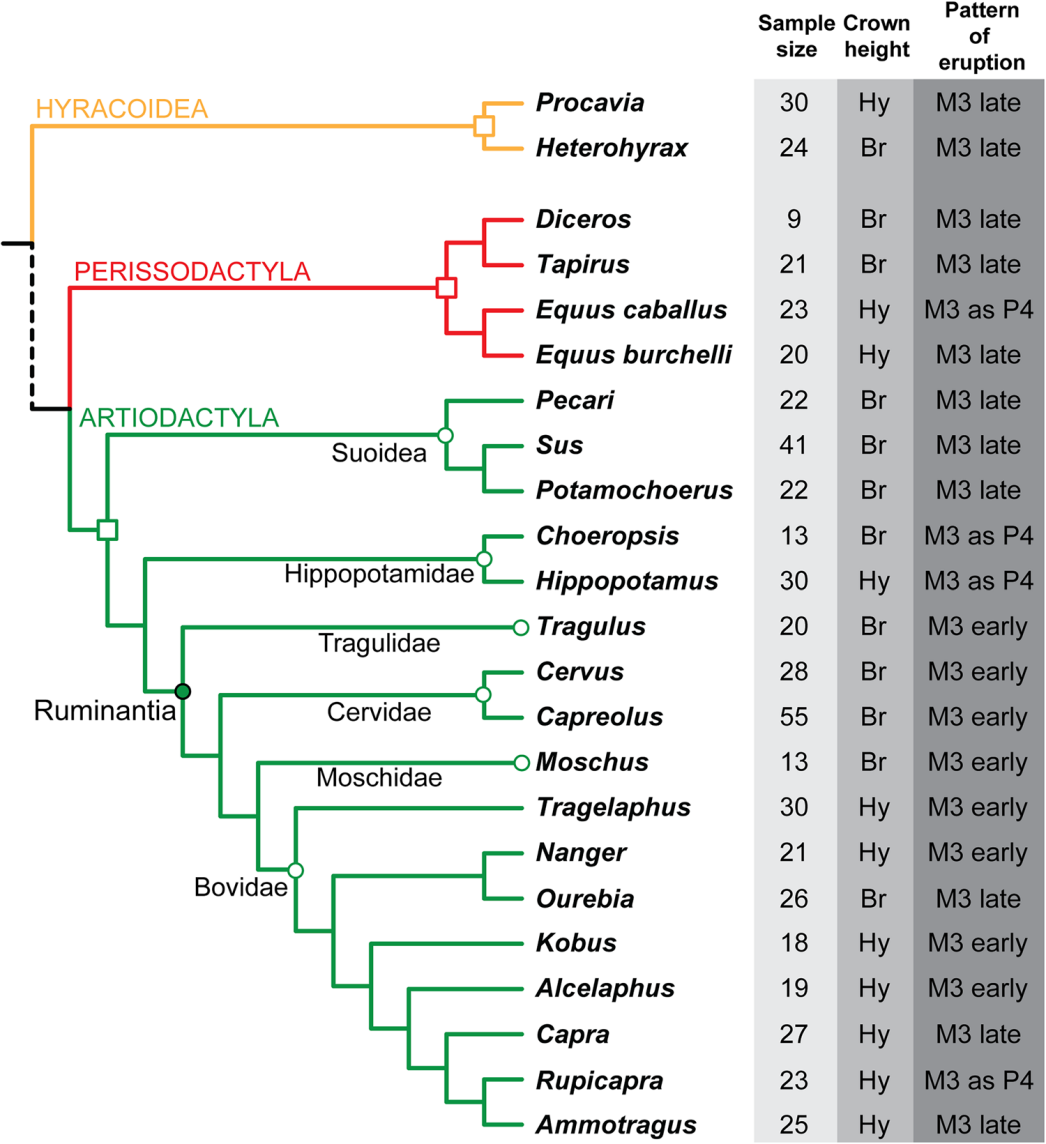
Phylogenetic relationships between the investigated ungulates (from Bibi 2013; Zurano et al. 2019), associated number of specimens, and dental characters. The dotted branch characterizes a more distant phylogenetic distance between Hyracoidea and Perissodactyla + Artiodactyla. Br, brachyodont; Hy, hypsodont.

Numerous comparative studies have focused on the shape of adjacent teeth or on the co‐variation between teeth proportions (Butler 1939; Freeman 1998; Kavanagh et al. 2007; Renaud et al. 2009; Hlusko et al. 2011; Evans et al. 2016). The jaw has also been frequently investigated from allometric or ontogenetic viewpoints (Cardini and Polly 2013; Marcy et al. 2016; Dubied et al. 2021), but rarely in relation to dental variations (e.g., Gould 1975; Fortelius 1985; Gomes Rodrigues et al. 2025). While some studies have considered both dental and jaw sizes in their analyses or discussion, few have analyzed the covariation in shape of these structures, which therefore remains poorly understood (Ford 1980; Monson et al. 2019; Billet and Bardin 2021; Clauss et al. 2022; Sadier et al. 2023). Here, we proposed to comparatively analyse the shape of the cheek teeth and palate in the course of mammalian ontogeny, encompassing both the replacement of premolars and the eruption of molars. Dental shape and size are influenced by a number of factors, including differential crown heights combined with wear. Premolar replacement and molar eruption also affect the shape and size of the whole dentition during ontogeny. To understand how the palate accommodates these changes, we focused on several ungulate clades (i.e., Artiodactyla, Perissodactyla and Hyracoidea) known for their diversity of cranial shapes and dental specializations in terms of crown height and eruption patterns (Figure 1; Janis and Fortelius 1988; Smith 2000; Monson and Hlusko 2018; Gomes Rodrigues et al. 2019). Using geometric morphometric analyses, we evaluated variation in shape and the integration of the teeth‐palate complex across ontogenetic stages, considering the degree of dental specializations (i.e., crown height, eruption pattern). The different patterns of variation and co‐variation were comparatively analysed in a wide range of ungulate species, to enhance our understanding of the mutual influence that dental and palatal units exert on each other during mammalian ontogeny.

## 2 Materials and Methods

### 2.1 Sample Composition

The ungulate database used in this study consists of mammal specimens stored at the Muséum National d'Histoire Naturelle in Paris (MNHN), complemented by specimens of wild boar (*Sus scrofa*) previously CT‐scanned for the DOMEXP project (https://archeozoo-archeobota.mnhn.fr/fr/anr-domexp-8878; Neaux et al. 2021, 2022; Cucchi et al. 2023). We analyzed 560 crania representing 23 ungulate species: two Procaviidae (Hyracoidea), two Equidae, one Rhinocerotidae, one Tapiridae (Perissodactyla), eight Bovidae, two Cervidae, two Hippopotamidae, two Suidae, one Moschidae, one Tayassuidae, and one Tragulidae (Artiodactyla, Figure 1; Supporting Information S1). We analyzed ontogenetic series involving juvenile to adult specimens (i.e., from specimens having only the first molar and deciduous premolars erupted, to others having all molars and permanent premolars erupted). We chose to focus on the upper jaw because it experiences less drastic mechanical constraints than the comparatively narrow lower jaw (Renvoisé et al. 2017). DPx stands for deciduous premolars, Px for permanent premolars, and Mx for molars, with x representing the locus of each upper tooth. The use of individual dental age stages (IDAS) as defined by Anders et al. (2011) did not seem relevant in our case, because the present ontogenetic series only covers three IDAS stages (IDAS 2, 3, and 4). We used instead six ontogenetic stages that cover shorter periods and are mainly based on dental eruption: 1—specimens with only M1 erupted, 2—with M2 erupting, 3—with M2 erupted, 4—with M3 erupting, 5—with M3 erupted and M1 moderately worn, and 6— with M3 erupted and M1 highly worn (i.e., occlusal surface removed). Three additional stages were also considered in the different analyses regarding premolars: deciduous premolars only, permanent premolars replacing deciduous premolars, permanent premolars only. We also considered the dental crown height, categorized as low‐crowned (brachyodont) or high‐crowned (hypsodont; Figure 1). The dental eruption pattern was categorized into three stages (see Monson and Hlusko 2018; Gomes Rodrigues et al. 2017a, 2019): M3 erupting before permanent premolars (M3 early), simultaneous eruption of M3 and P4 (M3 as P4), M3 erupting last (M3 late; Figure 1). Sexual dimorphism was not taken into account because information was generally missing for most species.

### 2.2 Geometric Morphometric Methods

**Figure 2:**
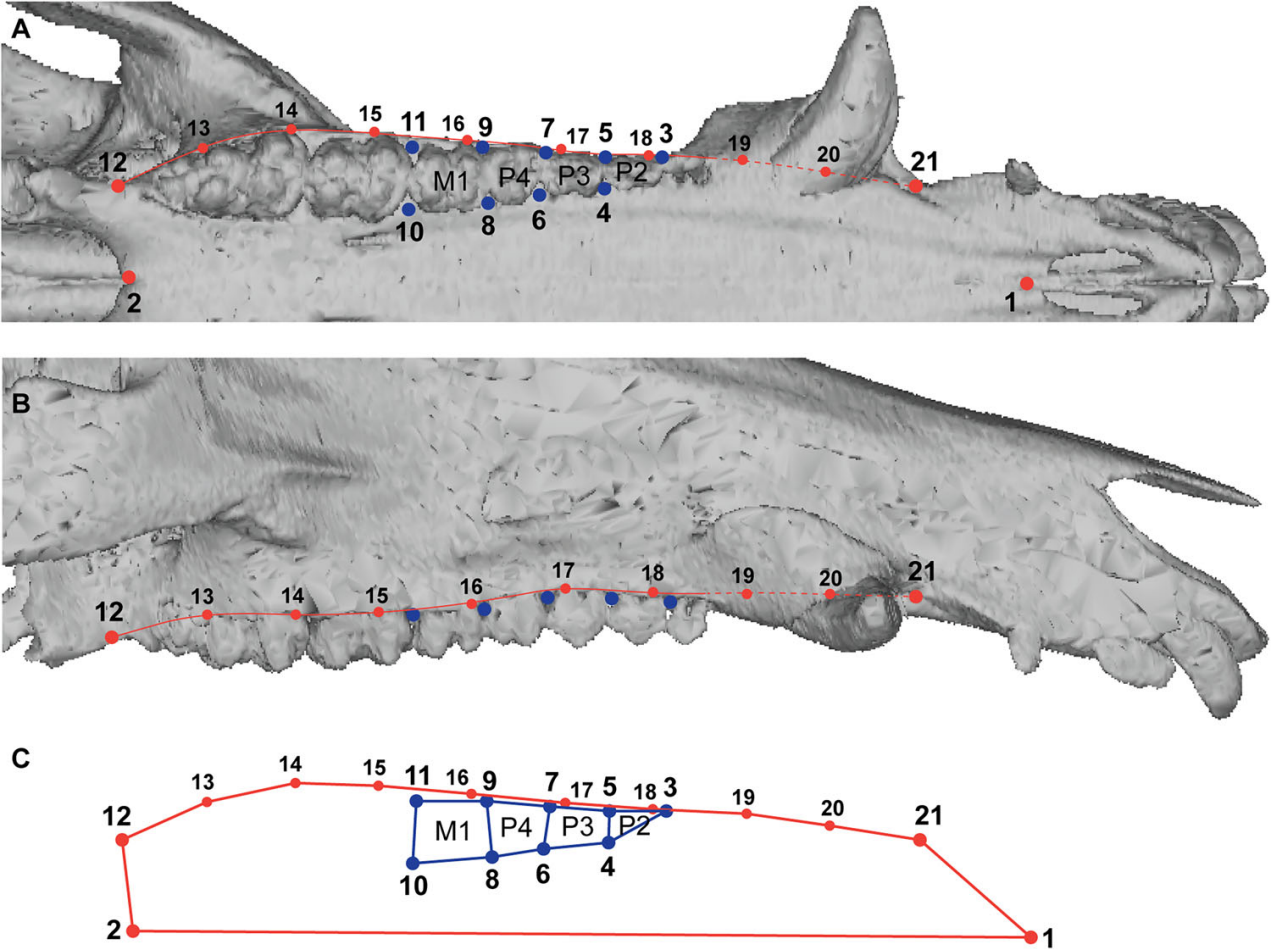
CT‐scan rendering of the cranium of a wild boar showing the location of the landmarks digitized. (A) palatal view, (B) lateral view, (C) Diagram showing the landmarks on the palate in red and on the dental row (P2‐M1) in blue. Not to scale.

We chose to focus on three premolar loci (P2, P3, and P4), which are present and replaced in all investigated specimens, in contrast to the first premolar locus (P1). We also included the first molar (M1), a tooth not replaced but already present in juvenile specimens, in contrast to the second and third molars (M2 and M3). The global shape of the right upper cheek teeth (i.e., P2, P3, P4, and M1) was quantified using 9 anatomical landmarks (Figure 2; Supporting Information S2, see Gomes Rodrigues et al. 2025). As some landmarks define the extreme mesial and distal points of each premolar locus, they also register the main shape changes from deciduous to permanent dentition. The shape of the right side of the palate was quantified using two anatomical landmarks defining the medial margin and two anatomical landmarks defining the labial margin of the maxilla. This was complemented with eight semi‐landmarks taken along a curve following this margin (Figure 2; Supporting Information S2; NB: the potential deformations associated with a large canine were not taken into account because they are not the focus of our study; this part of the curve was replaced by a straight line during semi‐landmarks resampling, see below). Digital data (landmark coordinates) were acquired using a Microscribe 3‐D digitizer for most species (G2X, Immersion Corporation, measurement error: 0.0001 mm). Only the right side of the cranium was investigated, but when it was damaged, the left side was used, in few cases (see Supporting Information S1), after being mirrored using the *mirror* function from the Morpho R package (Schlager 2023). For the wild boar, landmarks were digitized on reconstructed 3D meshes of crania using the “LANDMARK editor” (renamed as Checkpoint, https://www.stratovan.com/blog/idav-landmark-editor-checkpoint). The use of the microscribe to digitize a curve along the labial margin of the maxilla implies that the initial number of semi‐landmarks calculated varies from one specimen to another, depending on its size. As a result, the curve of each specimen was resampled using the function *digit.curves* from the geomorph R package (Adams et al. 2024) to obtain eight equidistant semi‐landmarks.

All configurations (sets of 21 landmarks; Supporting Information S3) were superimposed using the Procrustes method of generalized least squares superimposition (GLS scaled, translated, and rotated configurations so that the intralandmark distances were minimized) following the method used by Rohlf (1999) and Bookstein (1991). This superimposition was performed using the function *gpagen* (geomorph R package), which includes the sliding of semi‐landmarks so as to minimize the bending energy (Gunz and Mitteroecker 2013). The shape variation of the teeth‐palate complex was analyzed across ontogeny by principal components analyses (PCA) in each species, using the *gm.prcomp* function and *plotRefToTarget*, from the same package, for the visualization of shape deformations on each component. Then, two GLS were performed on subsamples, meaning cheek teeth and palate separately. These separate sets of procrustes coordinates were used in two block partial least square analyses. They allowed to evaluate the pattern of co‐variation between the cheek teeth (block A) and the palate (block B) for each species, using the function *two.b.pls* from the geomorph package, but visualization of shape deformation of teeth and palate on each single warp axis were produced using the function *deformGrid3d* from the Morpho package. Finally, ratios of palate centroid size were also calculated to quantify and compare the overall growth rate of the palate at the different stages of molar eruption (mean palate centroid size at *M1 erupted*/mean palate centroid size at *M3 erupted*; mean palate centroid size at *M2 erupted/*mean palate centroid size palate at *M3 erupted*).

## 3 Results

**Figure 3:**
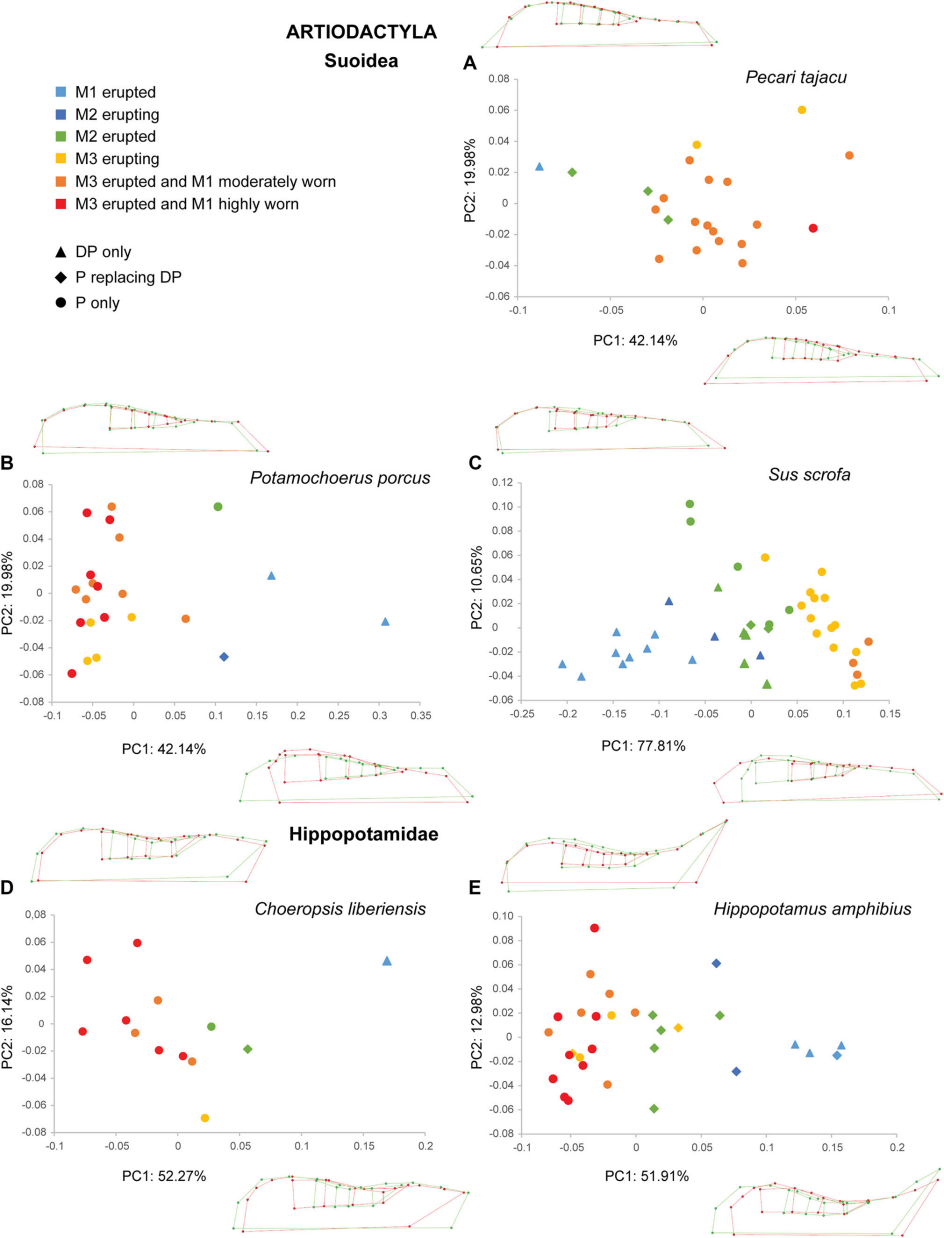
Graphs of PCA on the cranium for each suoid and hippopotamid species with virtual deformations (occlusal view) on the extreme side of each axis (green: deformation for the negative side, red: deformations for the positive side).

**Figure 4:**
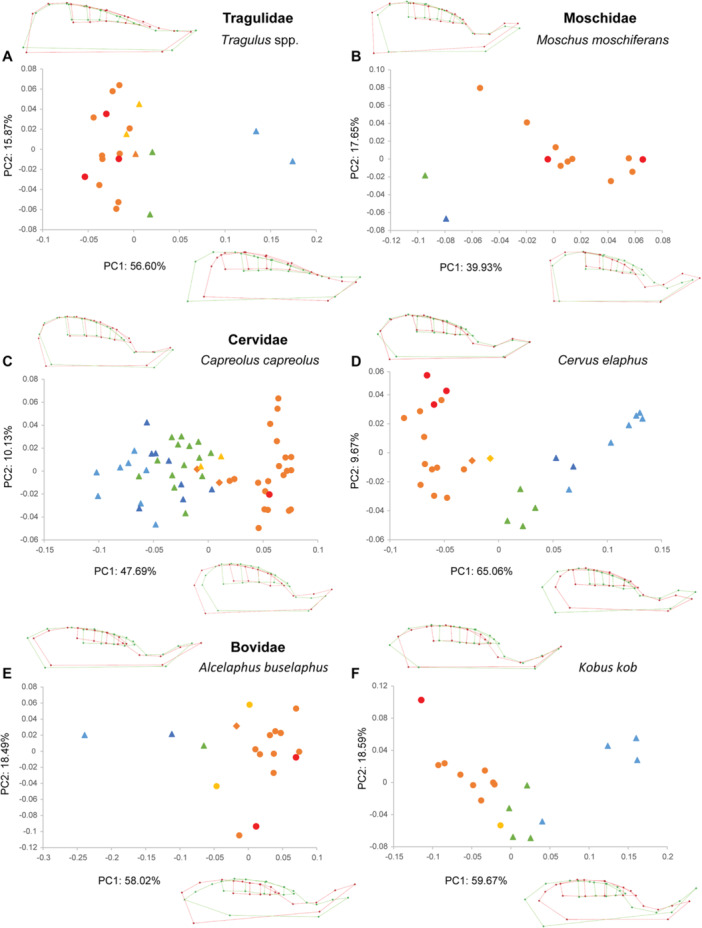
Graphs of PCA on the cranium for each ruminant species (part 1) with virtual deformations (occlusal view) on the extreme side of each axis (green: deformation for the negative side, red: deformations for the positive side). See caption on Figure 3.

**Figure 5:**
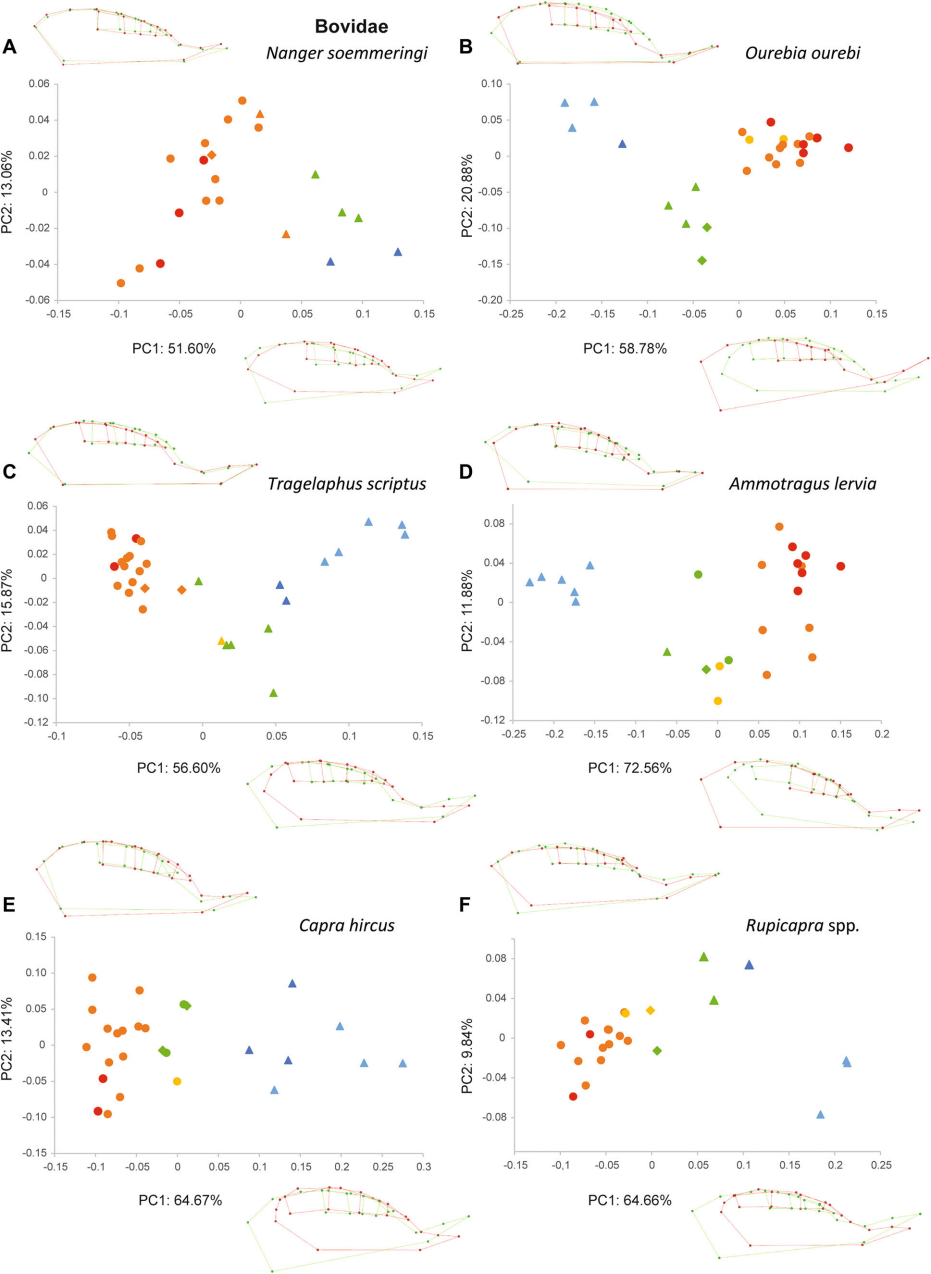
Graphs of PCA on the cranium for each ruminant species (part 2) with virtual deformations (occlusal view) on the extreme side of each axis (green: deformation for the negative side, red: deformations for the positive side). See caption on Figure 3.

**Figure 6:**
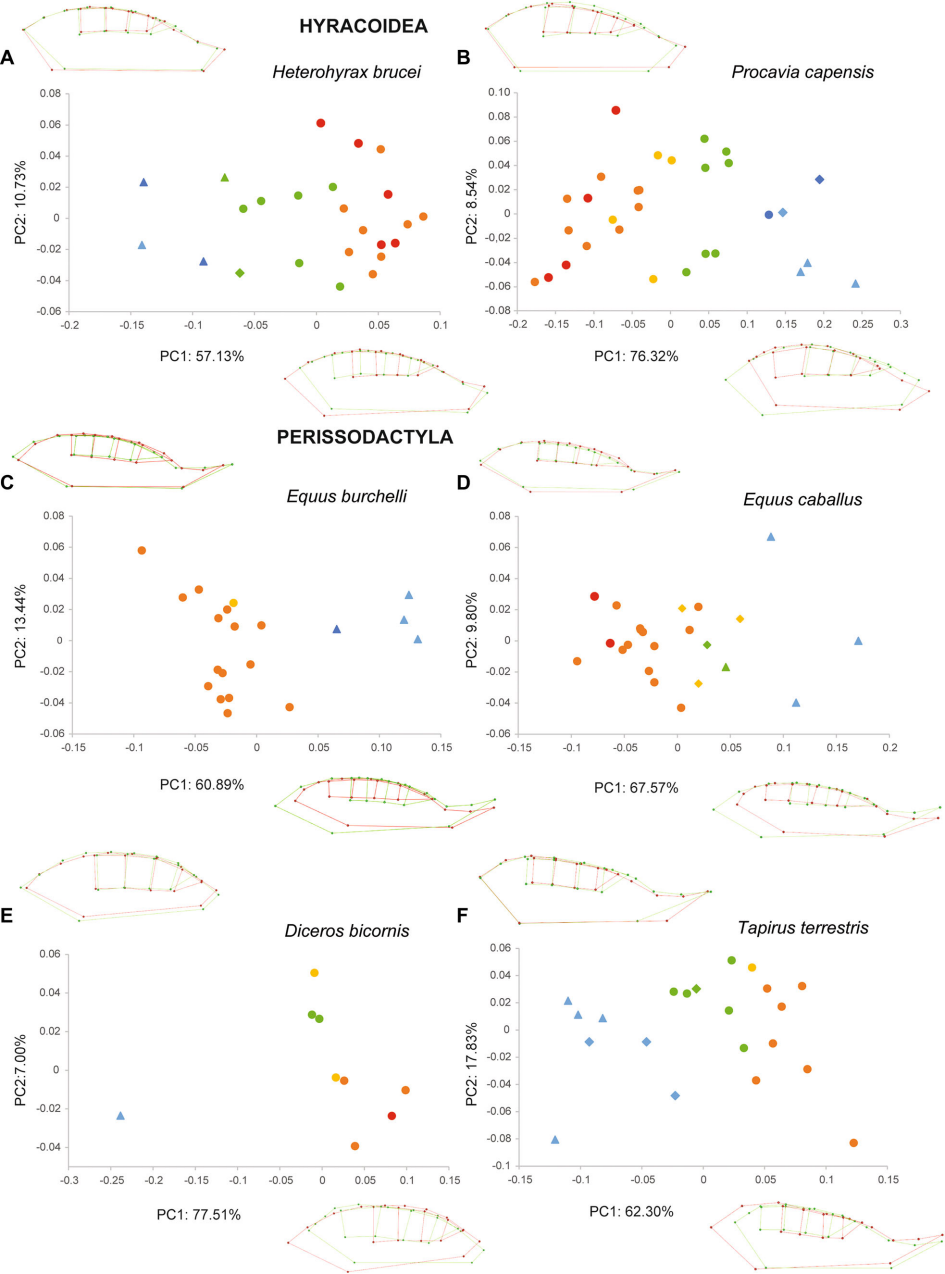
Graphs of PCA on the cranium for each hyracoid and perissodactyl species with virtual deformations (occlusal view) on the extreme side of each axis (green: deformation for the negative side, red: deformations for the positive side). See caption on Figure 3.

**Figure 7:**
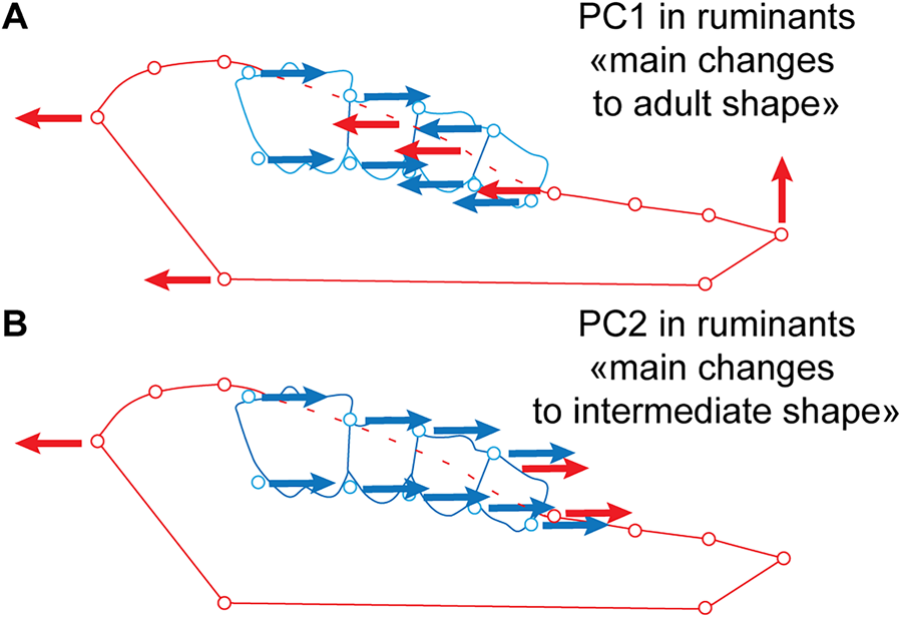
Schematic representation of the main deformations observed on PCA for ruminants. (A) PC1 and (B) PC2. Arrows indicate the orientation of the main deformations between juvenile and adult specimens. Dental deformations are displayed in blue, and those of the palate in red.

The PCAs performed within each species show similar patterns, especially on the first component (PC1, Figures 3, 4, 5, 6). In each analysis, PC1 generally explains more than 40% of the total variation, and PC2 less than 21%. PC3s (not shown here) and subsequent components have a more reduced contribution (< 10%) and are not informative enough to require a description. On PC1, the specimens are scattered according to ontogenetic stages: young specimens can be either on the positive or the negative side, and conversely for the old specimens. The extreme shape associated with young specimens corresponds to a short palate (except for *Pecari*; Figure 3A), with a slender and relatively elongated dental row (DP2‐M1), whereas adult specimens have a more elongated palate, including a more posterior distal edge, and a relatively shorter dental row (P2‐M1), more anterior on the palate, and including shorter teeth (Figure 7A). In most high‐crowned species (e.g., Figures 4, 5, and 6C,D), and in *Diceros* that shows strong interdental wear (Figure 6E), these extreme deformations are manifest, especially concerning the M1 which is highly elongated in young specimens, while it is shorter and more square‐shaped in older specimens. On the second component, the distribution of specimens does not show a clear patterning (as in following components), except in many ruminants (e.g., *Ammotragus*, *Cervus*, *Kobus*, *Ourebia*, *Rupicapra, Tragelaphus*; Figures 4 and 5). In these species, specimens of intermediate ontogenetic stages, especially those with M2 erupted, with DPs about to be replaced or being replaced, or with M3 erupting, plot on one extreme side of the axis (negative or positive, conversely to both younger and older specimens, especially those with highly worn teeth). This pattern is associated with a shape including a relatively more anterior dental row combined with a more anterior and more convex lateral part of the palate just posterior to the constriction, and more posterior and oblique edge of the maxilla (Figure 7B).

**Figure 8:**
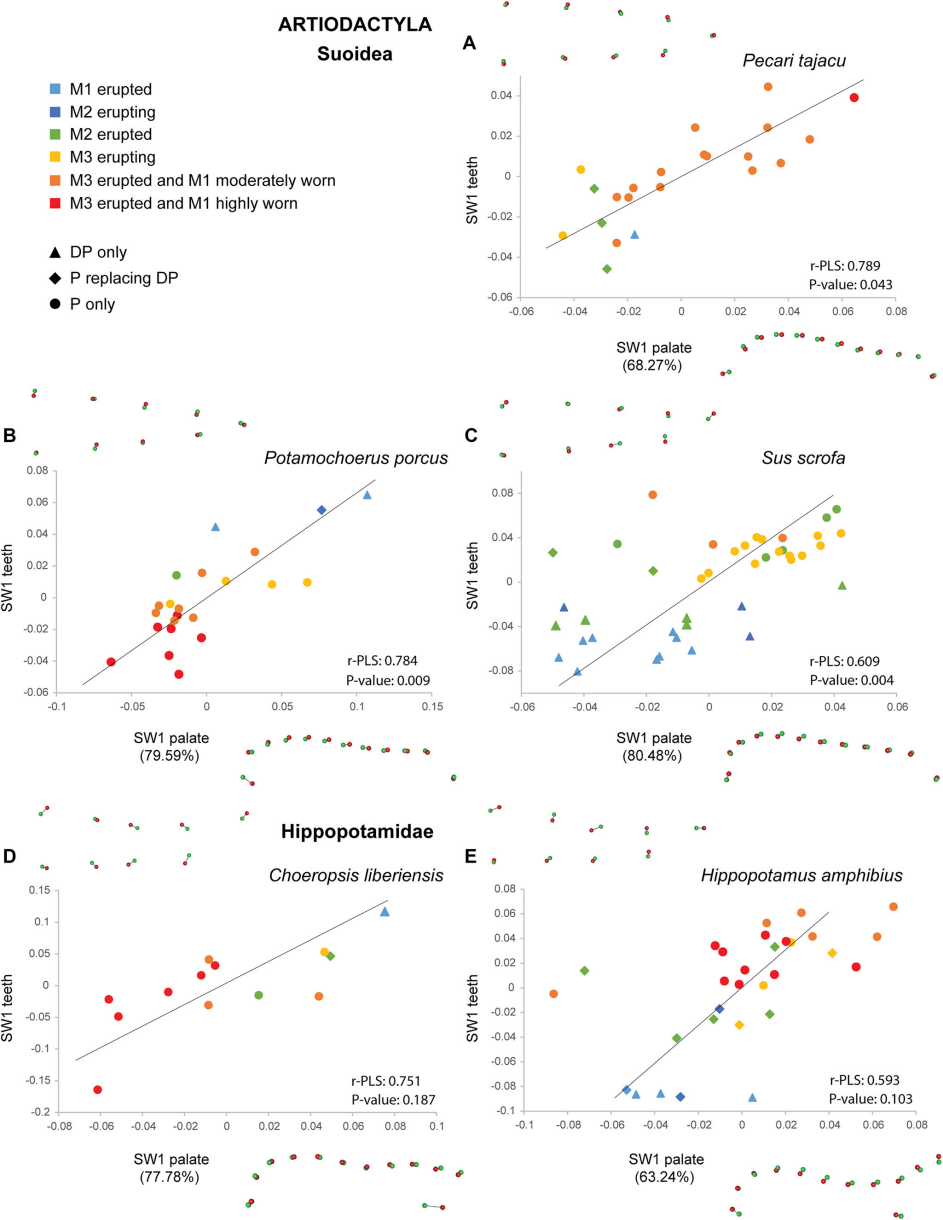
Graphs of 2B‐PLS between the palate and teeth for each suoid and hippopotamid species with virtual deformations (occlusal view) on the extreme side of each axis (green: deformation for the negative side, red: deformations for the positive side).

**Figure 9:**
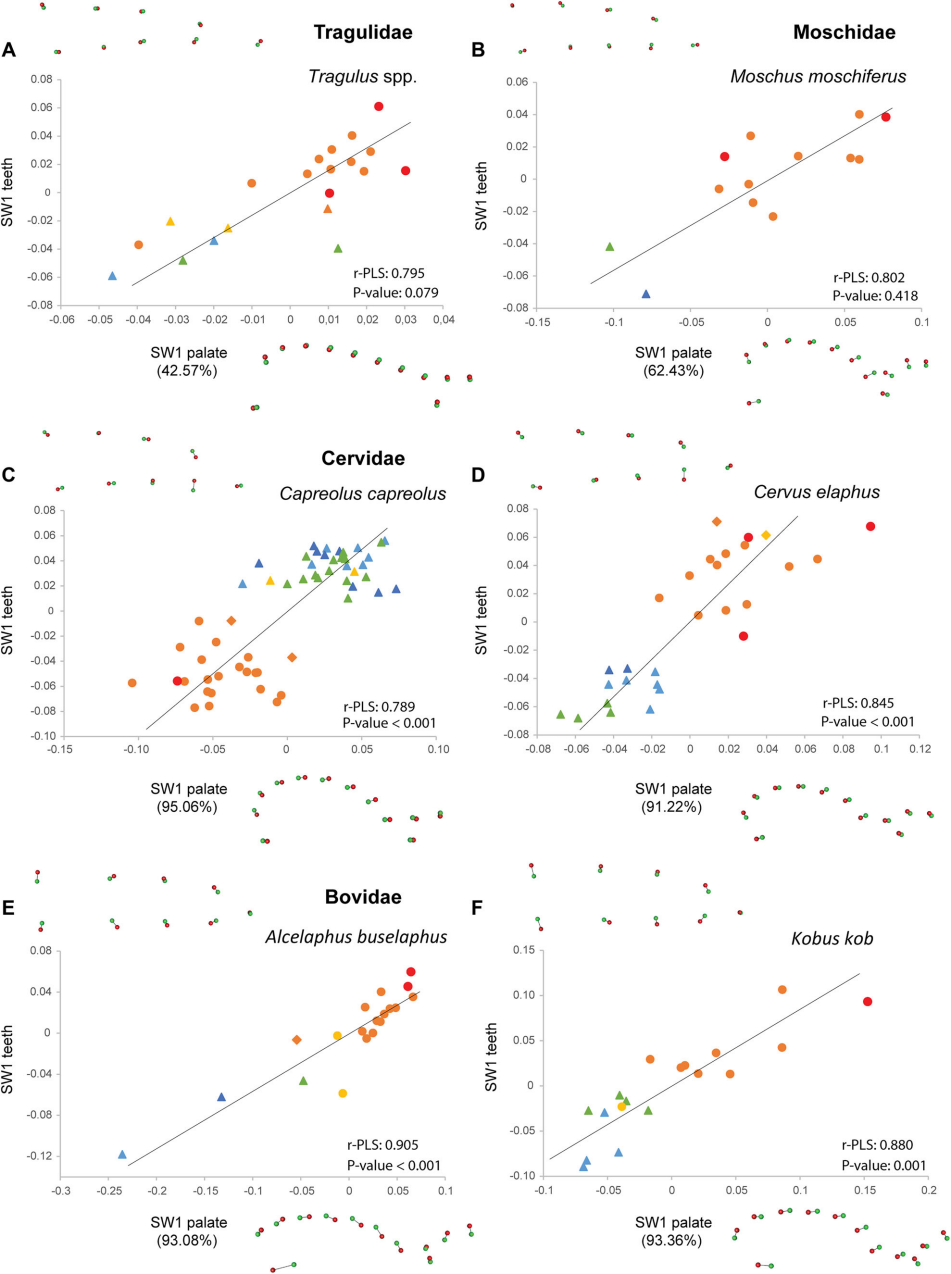
Graphs of 2B‐PLS between the palate and teeth for each ruminant species (part 1) with virtual deformations (occlusal view) on the extreme side of each axis (green: deformation for the negative side, red: deformations for the positive side). See caption on Figure 8.

**Figure 10:**
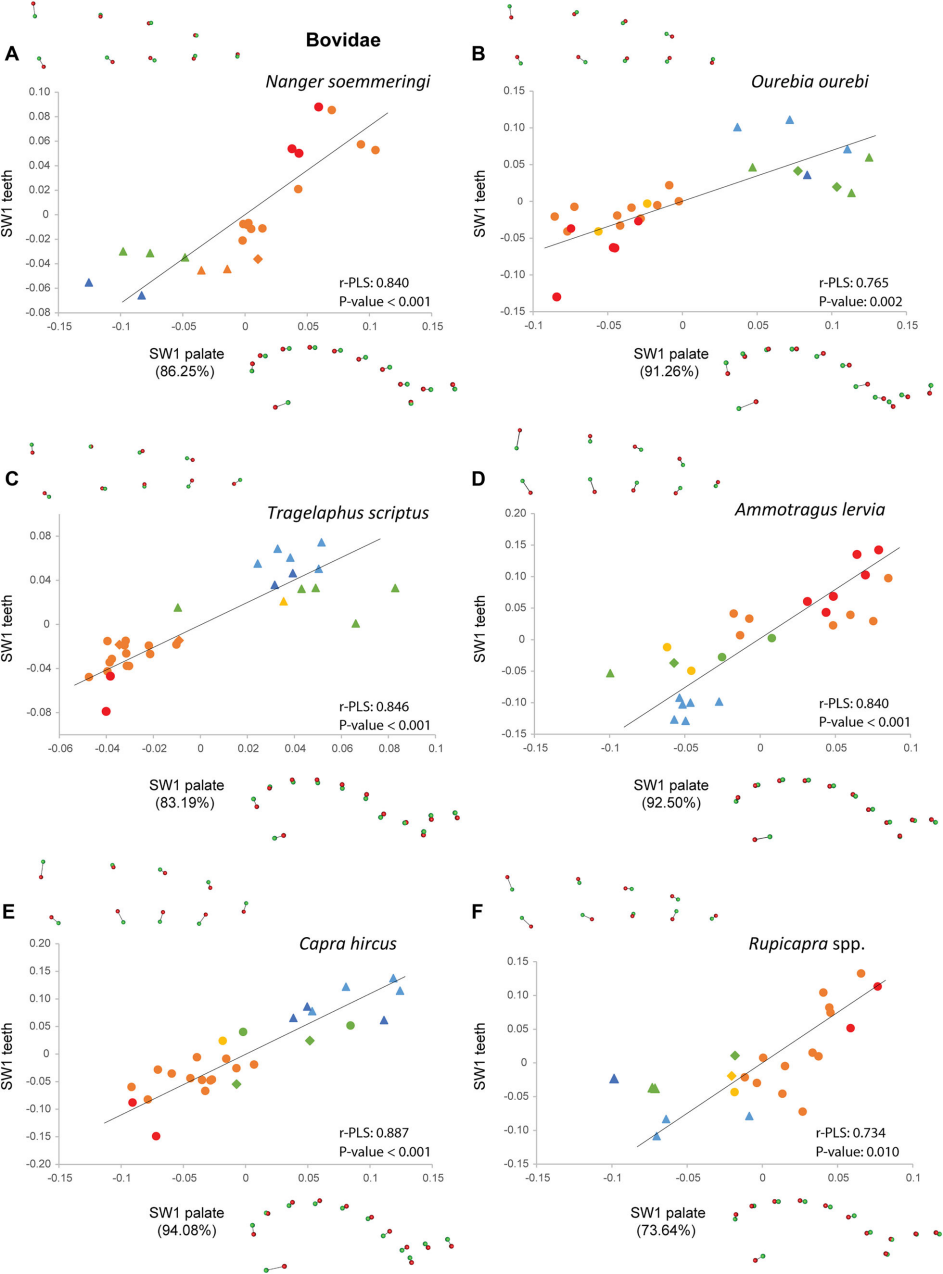
Graphs of 2B‐PLS between the palate and teeth for each ruminant species (part 2) with virtual deformations (occlusal view) on the extreme side of each axis (green: deformation for the negative side, red: deformations for the positive side). See caption on Figure 8.

**Figure 11:**
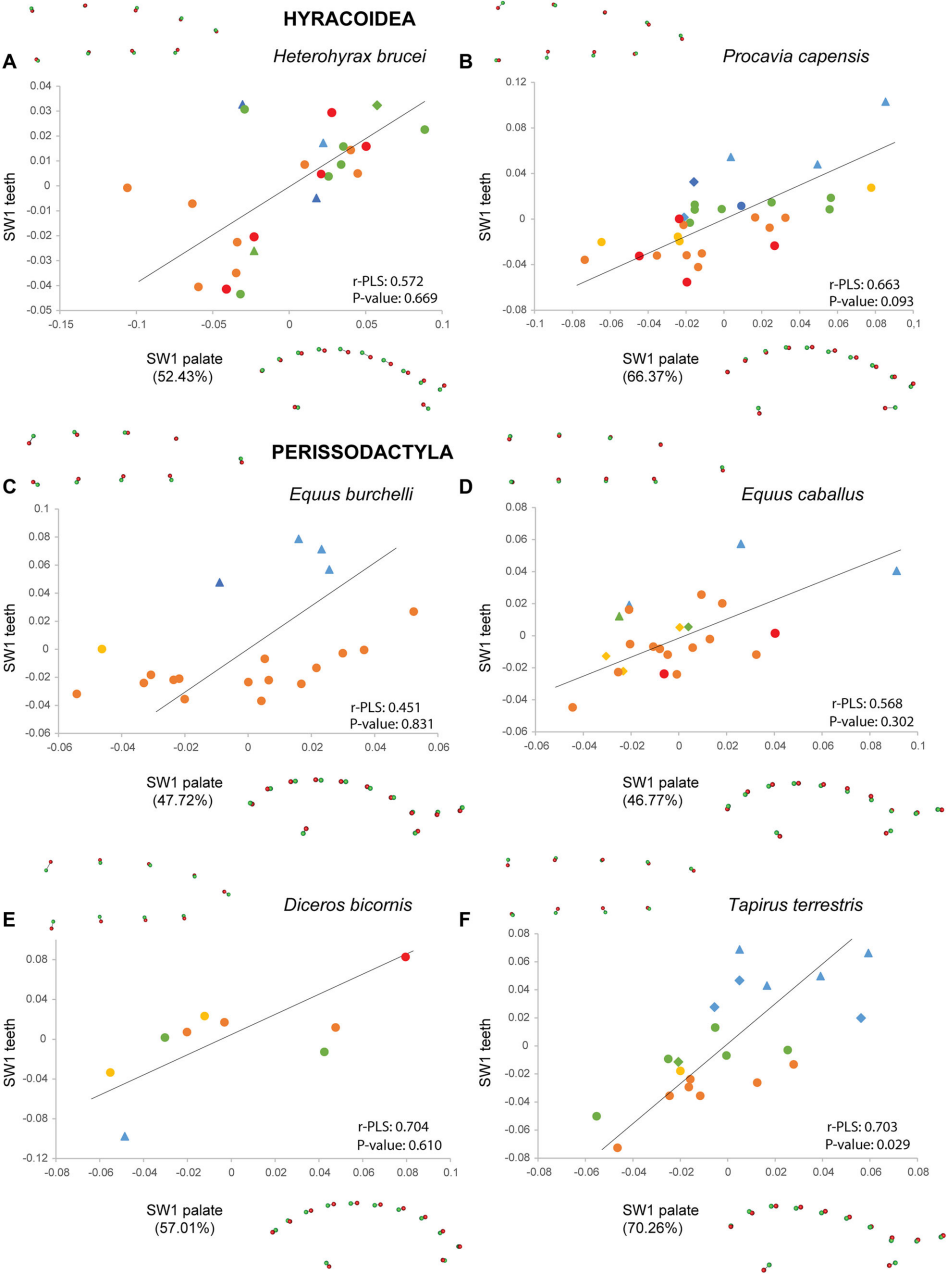
Graphs of 2B‐PLS between the palate and teeth for each hyracoid and perissodactyl species with virtual deformations (occlusal view) on the extreme side of each axis (green: deformation for the negative side, red: deformations for the positive side). See caption on Figure 8.

The analyses of co‐variation between palate and dental shape performed with two‐block PLS for each species also show shared patterns frequently associated with ontogenetic stages on the first single warp axis (SW1; Figures 8, 9, 10, 11), the following warp axes being poorly informative. Regressions between the SW1 of palate and teeth are significant for most ruminants, except for *Tragulus*, and *Moschus* despite their high r‐PLS (Figures 9 and 10), and whatever the dental crown height or eruption pattern. The regressions are also significant for suoids and *Tapirus*, but not for hippos, hyraxes, equids, and the rhino (Figures 8 and 11). In some species, a relatively high r‐PLS does not correspond to a significant regression due to their small sample size (~10 specimens, e.g., *Choeropsis*, *Diceros, Moschus*), while other species show marginally significant regressions (*p* value < 0.1, i.e., *Tragulus*, *Procavia*). In most species in which the covariation between the palate and teeth is significant or in which the r‐PLS is relatively high, the specimens are distributed along the regression line according to their ontogenetic stage. In these species, a slender dental row is most frequently associated with an anterior projection of the palate posterior to the constriction, and more anterior choanae, which correspond to the condition in juvenile specimens. Adult specimens, especially old specimens with worn teeth, present a combination of a posterior enlargement of the palate, more posterior choanae and a wider dental row, which are reminiscent of the deformations observed for adults in the PCAs (Figure 7A). These observations are less obvious in suoids, especially concerning the palate that shows only minor deformations (slightly more slender palate in adults, Figure 8A–C).

**Figure 12:**
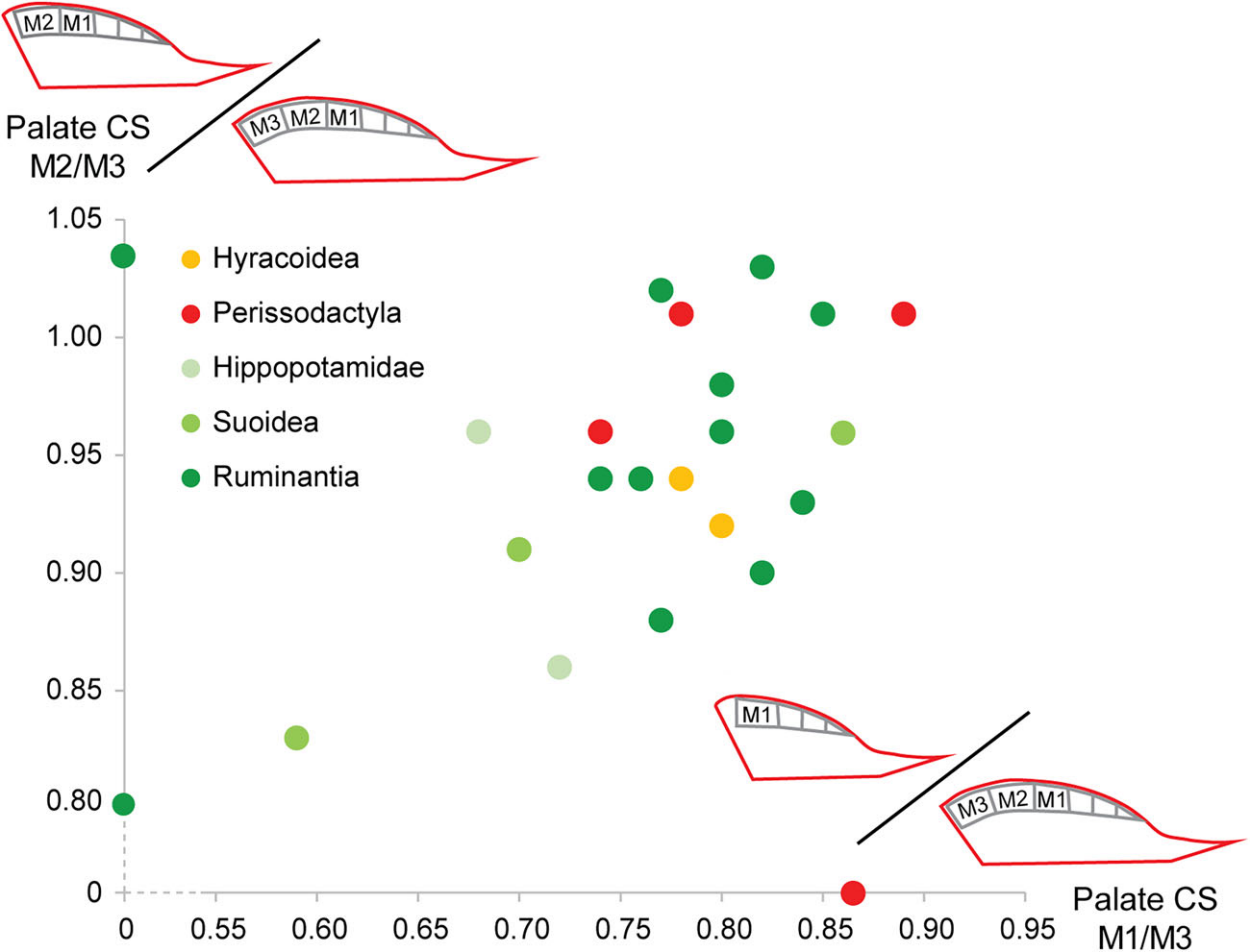
Biplot of the mean ratios of palate centroid size for M1 erupted vs. M3 erupted (CS palate M1/M3) vs. the mean ratios of palate centroid size for M2 erupted vs M3 erupted (CS palate M2/M3). Values equal to zero for a few species in the graph correspond to missing data.

Ratios of palate centroid size for *M1 erupted versus M3 erupted* (i.e., palate CS M1/M3) show that hippopotamids and suids have the lowest values (< 0.72), meaning that M1 erupts early compared to palatal growth in these taxa (x‐axis, Figure 12; Supporting Information S4). Conversely, Hyracoidea and most Perissodactyla have high values, as do most ruminants and *Pecari* (> 0.78). *Diceros, Tragulus, Cervus*, *Ammotragus and Capra* show intermediate values. Ratios of palate centroid size for *M2 erupted versus M3 erupted* (i.e., palate CS M2/M3) show less obvious discrepancies among taxa (y‐axis, Figure 12). It is only possible to mention that some taxa have values under 0.90, such as *Hippopotamus*, *Potamochoerus*, *Moschus*, and cervids, meaning M2 erupts early compared to bone growth in these taxa. Some Perissodactyla and ruminants even show values exceeding 1.00, such as *Tapirus*, *Equus*, *Ammotragus*, *Ourebia*, and *Alcelaphus*, meaning that sub‐adults have a surprisingly larger palate than adults. This might be explained by diverse size variations between different populations (e.g., *Equus*), or by the presence of only young adults (M3 just erupted; e.g., *Tapirus*), which minimize the size differences between adult and young specimens. It is important to mention that these different ratios include only one specimen for M1 eruption stage of some species (*Alcelaphus*, *Diceros*, *Choeropsis*, *Heterohyrax*, and *Pecari*), and for M2 eruption stage (*Alcelaphus*, *Moschus*, *Potamochoerus*, and *Pecari*; Supporting Information S4), meaning they should be considered cautiously.

## 4 Discussion

### 4.1 Differential Scaling of Teeth and Palate During Ontogeny

Despite the discrepancies in sample sizes, with some species represented by a low number of specimens (e.g., *Diceros*, *Choeropsis, Moschus*), and the potential lack of juvenile or adult specimens for some species (e.g., *Alcelaphus*, *Pecari*, *Sus*, *Tragulus*), several results are consistently repeated across species. The main limitation of our study is probably that some samples would benefit from more specimens at the early stages, as our results on centroid sizes suggest. However, this seems a minor issue when considering our main results: in each ungulate species analysed, the main component of shape variation of the teeth–palate complex is associated with a clear ontogenetic patterning. In all investigated species, juveniles exhibit an elongated dental row (DP2‐M1) compared to the palate, while the palate is more elongated with more posterior choanae, and the dental row (P2‐M1) is comparatively reduced in adults, according to the PCAs. Overall, our results thus suggest that a longer palate is associated with a relatively shorter dental row in adults (P2‐M1) versus juveniles (DP2‐M1) in many species (Figure 7A).

These results are not surprising, because the absolute size of teeth is fixed early and barely changes at a given locus after replacement (except for the influence of wear and crown height, see below), while the palate grows over a longer period to house the later erupting second and third molars (e.g., Fortelius 1985). The differential growth between these units therefore make teeth appear shorter at adult stages relative to the palate and this probably holds true relative to the overall cranial size as well. These findings are also reminiscent of an intraspecific allometric pattern observed for the whole dentition relative to skull size in some horse and cattle breeds, which exhibit smaller skulls and relatively longer tooth rows (Radinsky 1984; Clauss et al. 2022). This pattern is actually observed in many dwarf mammals (e.g., Gould 1975; Shea and Gomez 1988; Lister and Hall 2014) and a lower evolvability of tooth size versus cranial size is generally invoked to explain it (e.g., Fortelius 1985; Lister and Hall 2014; Clauss et al. 2022). As regards to the pattern observed during ontogeny, the underlying scaling mechanisms are likely to be activated early in the development of teeth compared to the skull (Shea and Gomez 1988; Christensen et al. 2023). Given the concordance of scaling patterns observed at the microevolutionary and ontogenetic levels, potential links between an early developmental activation of scaling mechanisms and a lower evolvability, as proposed for tooth size, might deserve further exploration.

Our Procrustes analysis, including both palate and teeth can give the misleading impression of changes in tooth size constrained by the palate (see PCA), but when palate and teeth are considered separately in different Procrustes analyses (see PLS), our results obviously reflect changes in tooth proportions not directly related to the palate. In adult and old specimens, we also observed less elongated teeth, which comparatively appear as wider, and this is likely associated to the dental wear gradient. This shape deformation is generally marked in many high‐crowned taxa (e.g., Gomes Rodrigues et al. 2017b), in which the occlusal design is strongly modified by wear and tends to be more square‐shaped. Inter‐dental wear also affects adjacent teeth, due to the biomechanical pressure (i.e., compressive forces) exerted by the last erupted molars on more anterior cheek teeth resulting in wearing out their contiguous crown. The most remarkable examples of inter‐dental wear are found among rhinocerotids (Hitchins 1978; Hullot et al. 2024). These progressive modifications of dental shape due to wear, present in both hypsodont and non‐hypsodont species, explain why the single‐generation M1 locus can also undergo changes in outline across ontogenetic stages (Hullot et al. 2024), even if these changes are not expected considering that this tooth is not replaced. A study involving only the adult stages and including all molars (i.e., M1‐M3) in the landmark data set would have enabled a more in‐depth discussion of the impact of dental wear on the shape of all cheek teeth.

### 4.2 A Distinctive Growth Pattern of the Teeth–Palate Complex in Ruminants

Our study also reveals that the majority of ruminants display a transitional pattern at intermediate ontogenetic stages (i.e., mainly “M2 erupted” stage), notably characterized by a more anterior dental row and inflated lateral part of the palate posterior to the constriction of the rostrum. This pattern cannot be explained by a distinctive height of dental crown, given that it occurs in both low‐crowned and high‐crowned ruminant species, and it also occurs in species having late eruption or early eruption of M3. Nor can it be associated with a specific timing of M2 eruption, inasmuch as we do not observe any clear difference between ruminants and other ungulates according to our ratio (cf. palate CS M2/M3). The peculiarity of artiodactyls, and especially some ruminants which show such specific pattern in intermediate ontogenetic stages, might lie in a potential accelerated timing of ossification of their jaw which could depart from most mammals (Koyabu 2013) or in their permanent dentition erupting well after sexual maturity (Asher and Olbricht 2009), meaning probably late in relation to jaw growth. Such assumptions are, however, not supported by our estimates of the growth ratio of the palate of most ruminants, in relation to dental eruption, which is as fast in ruminants as in perissodactyls and hyracoids (cf. palate CS M1/M3). Nonetheless, this transient shape pattern could simply correspond to a mesial shift of the dentition, meaning a differential bony growth in the rear of the cheek tooth array that causes its progressive forward displacement, *sensu* Lentle and Hume (2010). It could also be possible that the lateral margin of the palate may undergo a temporary bulging to accommodate the crypts of incoming permanent premolars. However, the reason why these phenomena would be more pronounced in ruminants than in other ungulates remains unclear.

The results of co‐variation analyses tend to corroborate the hypothesis of a specific pattern of growth of the teeth–palate complex in ruminants, since they show the highest r‐PLS. This highlights the strong integration between their palate and dental row, which might be related to their rapid pace of palate growth (and see life history traits in Sibly and Brown 2007) and fast dental eruption of the whole dentition (Monson and Hlusko 2018), the most widespread conditions in extant ruminants, compared to suoids and hippopotamids. It is assumed that this fast growth associated with a fast dental eruption is plesiomorphic in artiodactyls (Gomes Rodrigues et al. 2019). The combined fast pace of growth and dental eruption may generate specific constraints regarding the space available for the dentition within the jaw, which, in turn, may foster a more synchronised development, and thus a higher integration between those two anatomical elements in ruminants. However, this assumption needs to be more thoroughly investigated, given that caprines (*Ammotragus*, *Capra*, and *Rupicapra*) and *Ourebia* present a delayed eruption of the whole dentition (i.e., M3 erupting last), but still have a high co‐variation between teeth and palate. Nonetheless, the delayed eruption of last molars is a character state only “recently” acquired in caprines, as in *Ourebia* (i.e., less than 15 Ma; Monson and Hlusko 2018; Zurano et al. 2019), contrary to suids and hippopotamids (i.e., more than 35 Ma; Gomes Rodrigues et al. 2019). Despite their later molar eruption, caprines and *Ourebia* might present a phylogenetically conserved pattern for the teeth‐palate growth, which could in turn explain why structural constraints are quite similar for the diverse ruminant jaws despite the different dental eruption patterns. Including the last molars in the landmark data set (i.e., M2‐3), focusing on sub‐adult and adult specimens only, would have addressed this issue. However, this additional analysis might minimise the distinctive pattern observed in ruminants considering the entire cheek dentition, and in the absence of young stages.

### 4.3 Dental Accommodation as a Result of Structural Constraints and Adjustments

Results on ruminants suggested that inherited paces of both palate growth and dental eruption could influence the strength of structural constraints on the dentition in relation to the space available within the jaw. Hyracoids, hippopotamids, perissodactyls and some suoids generally exhibit more heterogeneous patterns of growth and covariation than ruminants. Among these, some species show high r‐PLS and significant to near significant regression (e.g., *Choeropsis*, *Diceros*, *Pecari*, *Potamochoerus*, *Procavia*, *Tapirus*), meaning that structural constraints might be high in these clades, although less than in ruminants. Suoids and hippos present a slow growth of the palate compared to molar eruption (cf. palate CS M1/M3), and their M3 eruption is delayed compared to permanent premolars (Monson and Hlusko 2018; Gomes Rodrigues et al. 2019). In suoids, as well as in other nonruminant ungulates, such an extended time for the last molar eruption including M3, but also M2 in some taxa (e.g., *Procavia*, *Hippopotamus*) also implies a later mineralization of these teeth. Consequently, it might also imply a later biomechanical pressure on the anterior part of the dentition, potentially relaxing the constraints on the coordinated growth of the teeth–palate complex. This could explain why the palate and dental row shapes of some of these species tend to covary less, even if the pace of the palate growth is elevated (e.g., similar values in perissodactyls and hyracoids as in ruminants). Regarding the comparatively low integration between the jaw and cheek teeth, it is indeed possible that the late eruption of molars characterizing most of suoids, hippopotamids, perissodactyls and hyracoids very early during their evolution (Böhmer et al. 2016; Asher et al. 2017; Domingo et al. 2018; Gomes Rodrigues et al. 2019) leaves more room for dental accommodation on the palate during ontogeny (e.g., Veitschegger and Sánchez‐Villagra 2016; Marchiori et al. 2019).

Apart from structural constraints, structural adjustments represent another putative mechanism of dental accommodation. Structural adjustments have previously been suggested in the cases of the evergrowing and/or drifting teeth, and they correspond to the hypothesized trade‐off between dental growth and palate growth, including the effect of wear, which allows the whole dentition to always fit the space available in the jaw (Lentle and Hume 2010; Gomes Rodrigues et al. 2017b). The idea of structural adjustments is also supported in old specimens with worn and modified dental shapes presenting stronger palatal deformations than most other adults. These adjustments probably result from bone remodelling, not growth, which allows the preservation of the integrity of the dental alveoli in regulating the alveolar bone shape surrounding the teeth (Henneman et al. 2008).

## 5 Conclusion

The high degree of integration of the dentition and the palate observed in the course of ontogeny in several ungulates suggests a coordinated growth involving structural constraints and adjustments, which is in line with the non‐independent relationships already noted for teeth and their surrounding bone (e.g., Grüneberg 1937; Wise and King 2008; Lungová et al. 2011; Renvoisé et al. 2017). In our sample, the integration is mostly significant in ruminants, which are mostly characterized by a putative combination of an inherited fast growth and early eruption of M3 (Gomes Rodrigues et al. 2019). From this perspective, a high degree of integration between jaw and teeth might also be a plesiomorphic pattern in early diverging placentals, since their early representatives may have experienced rapid growth (Hurum and Chinsamy‐Turan 2012; Funston et al. 2022). In other ungulates with late eruption of M3, this integration is somewhat weaker, which suggests relaxed structural constraints on the teeth‐palate complex. Consequently, a deep‐rooted dental eruption pattern might have a greater influence on the teeth‐palate shape, than variations of dental crown height. A recent longitudinal study in captive wild boars (Gomes Rodrigues et al. 2025) also noted that the timing of dental eruption might influence the place of the dentition within the jaw during ontogeny. Although our results may suggest a potential link between the pace of growth, dental eruption and integration within the teeth‐palate complex, further investigation is required to determine the precise nature of this potential association in these taxa, the underlying causal mechanisms and its generalizability to other mammalian species. Further explorations of the dental and bone ontogenetic traits of ungulates in relation to their growth strategies (i.e., dental growth and eruption, skull growth, body growth, life history traits) and feeding habits are warranted to better estimate the extrinsic and extrinsic factors influencing the shape and growth of the jaw.

## Ethics Statement

This article does not contain any studies with human participants or animals performed by any of the authors.

## Conflicts of Interest

The authors declare no conflicts of interest.

## 1 Peer Review

The peer review history for this article is available at https://www.webofscience.com/api/gateway/wos/peer-review/10.1002/jez.b.23321.

## Supporting information


**Online resource 1:** List of specimens investigated in this study, including information on the dental eruption stage.

**Online resource 2:** List of landmarks used in this study. Mesial and labial sides of cheek teeth correspond to their anterior and posterior parts, respectively.

**Online resource 3:** Landmark coordinates.

**Online resource 4:** Ratios of palate centroid size at different stages of molar eruption.

## Data Availability

The data set generated and analyzed during the current study (i.e., Landmark coordinates) is available in Supporting Information S3.
