# Supplementary material for: Accommodation of Dental Variations During Jaw Growth in Ungulate Mammals
Source: J Exp Zool B Mol Dev Evol. 2025 Aug 5;344(8):487–504. doi: 10.1002/jez.b.23321 (PMC12626908; doi:10.1002/jez.b.23321)
Supplement: Supplementary file 1 — Online resource 1: List of specimens investigated in this study, including information on the dental eruption stage. [file JEZ-344-487-s001.docx]

**Online resource 1**. List of specimens investigated in this study, including information on the dental eruption stage. For “Specimen”, SSN means without number (“sans numéro” in french), and (L) indicates that the left side was used (and then mirrored). For “Dental eruption stage”, P2-4 M1-3w corresponds to old specimens with “w” meaning worn. In brackets: erupting teeth.

| Family | Species | Specimen | Dental eruption stage |
| --- | --- | --- | --- |
| Bovidae | *Alcelaphus buselaphus* | MNHN-ZM-AC-1912-525 | DP2-4 M1 |
|  | *Alcelaphus buselaphus* | MNHN-ZM SSN1 | DP2-4 M1-(2) |
|  | *Alcelaphus buselaphus* | MNHN-ZM-AC-1909-314 | DP2-4 M1-2 |
|  | *Alcelaphus buselaphus* | MNHN-ZM-AC-1945-128 (L) | DP2-4 M1-3 |
|  | *Alcelaphus buselaphus* | MNHN-ZM-2009-420 | DP2-4 M1-3 |
|  | *Alcelaphus buselaphus* | MNHN-ZM-AC-1945-129 | P2-(4)-M1-3 |
|  | *Alcelaphus buselaphus* | MNHN-ZM-AC-1928-52 | P2-4 M1-3 |
|  | *Alcelaphus buselaphus* | MNHN-ZM-AC-1945-125 | P2-4 M1-3 |
|  | *Alcelaphus buselaphus* | MNHN-ZM-AC-1945-126 | P2-4 M1-3 |
|  | *Alcelaphus buselaphus* | MNHN-ZM-AC-1945-127 | P2-4 M1-3 |
|  | *Alcelaphus buselaphus* | MNHN-ZM-AC-1945-130 | P2-4 M1-3 |
|  | *Alcelaphus buselaphus* | MNHN-ZM-AC-1945-131 | P2-4 M1-3 |
|  | *Alcelaphus buselaphus* | MNHN-ZM-AC-1945-132 | P2-4 M1-3 |
|  | *Alcelaphus buselaphus* | MNHN-ZM-AC-1945-133 | P2-4 M1-3 |
|  | *Alcelaphus buselaphus* | MNHN-ZM-2007-1361 | P2-4 M1-3 |
|  | *Alcelaphus buselaphus* | MNHN-ZM-2009-417 | P2-4 M1-3 |
|  | *Alcelaphus buselaphus* | MNHN-ZM SSN2 (1962) | P2-4 M1-3 |
|  | *Alcelaphus buselaphus* | MNHN-ZM-AC-1918-46 | P2-4 M1-3w |
|  | *Alcelaphus buselaphus* | MNHN-ZM-2017-2722 | P2-4 M1-3w |
| Bovidae | *Ammotragus lervia* | MNHN-ZM-AC-1861-1 | DP2-4 M1 |
|  | *Ammotragus lervia* | MNHN-ZM-AC-1903-5 | DP2-4 M1 |
|  | *Ammotragus lervia* | MNHN-ZM-AC-1922-60 | DP2-4 M1 |
|  | *Ammotragus lervia* | MNHN-ZM-AC-1906-126 | DP2-4 M1 |
|  | *Ammotragus lervia* | MNHN-ZM-AC-1903-6 | DP2-4 M1 |
|  | *Ammotragus lervia* | MNHN-ZM-AC-1879-18 | DP2-4 M1 |
|  | *Ammotragus lervia* | MNHN-ZM-AC-1897-73 | P(2-4) M1-2 |
|  | *Ammotragus lervia* | MNHN-ZM-AC-1945-141 | P(2)-DP3-4 M1-2 |
|  | *Ammotragus lervia* | MNHN-ZM-AC-1967-169 | DP2-4 M1-2 |
|  | *Ammotragus lervia* | MNHN-ZM-AC-1918-43 | DP2-4 M1-2 |
|  | *Ammotragus lervia* | MNHN-ZM-AC-1937-61 | P2-4 M1-(3) |
|  | *Ammotragus lervia* | MNHN-ZM-AC-2000-394 | P2-4 M1-(3) |
|  | *Ammotragus lervia* | MNHN-ZM-AC-1909-144 | P2-4 M1-3 |
|  | *Ammotragus lervia* | MNHN-ZM-AC-1930-241 | P2-4 M1-3 |
|  | *Ammotragus lervia* | MNHN-ZM-AC-1945-92 | P2-4 M1-3 |
|  | *Ammotragus lervia* | MNHN-ZM-AC-1967-34 | P2-4 M1-3 |
|  | *Ammotragus lervia* | MNHN-ZM-AC-1891-954 | P2-4 M1-3 |
|  | *Ammotragus lervia* | MNHN-ZM-2010-643 | P2-4 M1-3 |
|  | *Ammotragus lervia* | MNHN-ZM-2007-1325 | P2-4 M1-3 |
|  | *Ammotragus lervia* | MNHN-ZM-AC-1889-139 | P2-4 M1-3w |
|  | *Ammotragus lervia* | MNHN-ZM-AC-1913-165 | P2-4 M1-3w |
|  | *Ammotragus lervia* | MNHN-ZM-AC-1919-15 | P2-4 M1-3w |
|  | *Ammotragus lervia* | MNHN-ZM-AC-1923-2238 | P2-4 M1-3w |
|  | *Ammotragus lervia* | MNHN-ZM-AC-1947-72 | P2-4 M1-3w |
|  | *Ammotragus lervia* | MNHN-ZM-AC-1889-311 | P2-4 M1-3w |
| Bovidae | *Capra hircus* | MNHN-ZM-AC-1845-238 | DP2-4 M1 |
|  | *Capra hircus* | MNHN-ZM-AC-1908-211 | DP2-4 M1 |
|  | *Capra hircus* | MNHN-ZM-AC-1871-426 | DP2-4 M1 |
|  | *Capra hircus* | MNHN-ZM-AC-BVI-153 | DP2-4 M1 |
|  | *Capra hircus* | MNHN-ZM-AC-1902-398 | DP2-4 M1-(2) |
|  | *Capra hircus* | MNHN-ZM-AC-1884-2126 | DP2-4 M1-(2) |
|  | *Capra hircus* | MNHN-ZM SSN1 | DP2-4 M1-(2) |
|  | *Capra hircus* | MNHN-ZM-AC-1900-46 | P(2)-DP3-4 M1-2 |
|  | *Capra hircus* | MNHN-ZM-AC-1907-41 | P(2)-DP3-4 M1-2 |
|  | *Capra hircus* | MNHN-ZM-AC-A12104 | P2-4 M1-2 |
|  | *Capra hircus* | MNHN-ZM-AC-A12059 | P2-4 M1-2 |
|  | *Capra hircus* | MNHN-ZM-AC-A12094 | P2-4 M1-(3) |
|  | *Capra hircus* | MNHN-ZM-AC-1903-138 | P2-4 M1-3 |
|  | *Capra hircus* | MNHN-ZM-AC-1905-198 | P2-4 M1-3 |
|  | *Capra hircus* | MNHN-ZM-AC-1969-252 | P2-4 M1-3 |
|  | *Capra hircus* | MNHN-ZM-AC-1879-175 | P2-4 M1-3 |
|  | *Capra hircus* | MNHN-ZM-AC-1843-123 | P2-4 M1-3 |
|  | *Capra hircus* | MNHN-ZM-AC-1929-51 | P2-4 M1-3 |
|  | *Capra hircus* | MNHN-ZM-AC-1908-121 | P2-4 M1-3 |
|  | *Capra hircus* | MNHN-ZM-AC-1884-2098 | P2-4 M1-3 |
|  | *Capra hircus* | MNHN-ZM-AC-1906-12 | P2-4 M1-3 |
|  | *Capra hircus* | MNHN-ZM-AC-1902-471 | P2-4 M1-3 |
|  | *Capra hircus* | MNHN-ZM-AC-1884-2084 | P2-4 M1-3 |
|  | *Capra hircus* | MNHN-ZM-AC-1871-318 | P2-4 M1-3 |
|  | *Capra hircus* | MNHN-ZM-AC-A12058 | P2-4 M1-3 |
|  | *Capra hircus* | MNHN-ZM-AC-1903-80 | P2-4 M1-3w |
|  | *Capra hircus* | MNHN-ZM-AC-1902-547 | P2-4 M1-3w |
| Bovidae | *Capreolus capreolus* | MNHN-ZM-AC-1993-154 | DP2-4 M1 |
|  | *Capreolus capreolus* | MNHN-ZM-AC-1993-166 | DP2-4 M1 |
|  | *Capreolus capreolus* | MNHN-ZM-AC-1993-168 | DP2-4 M1 |
|  | *Capreolus capreolus* | MNHN-ZM-AC-1993-170 | DP2-4 M1 |
|  | *Capreolus capreolus* | MNHN-ZM-AC-1993-171 | DP2-4 M1 |
|  | *Capreolus capreolus* | MNHN-ZM-AC-1993-172 | DP2-4 M1 |
|  | *Capreolus capreolus* | MNHN-ZM-AC-1993-181 | DP2-4 M1 |
|  | *Capreolus capreolus* | MNHN-ZM-AC-1993-192 | DP2-4 M1 |
|  | *Capreolus capreolus* | MNHN-ZM-AC-1993-190 | DP2-4 M1-(2) |
|  | *Capreolus capreolus* | MNHN-ZM-AC-1993-195 | DP2-4 M1-(2) |
|  | *Capreolus capreolus* | MNHN-ZM-AC-1993-200 | DP2-4 M1-(2) |
|  | *Capreolus capreolus* | MNHN-ZM-AC-1993-201 | DP2-4 M1-(2) |
|  | *Capreolus capreolus* | MNHN-ZM-AC-1993-207 | DP2-4 M1-(2) |
|  | *Capreolus capreolus* | MNHN-ZM-AC-1993-215 | DP2-4 M1-(2) |
|  | *Capreolus capreolus* | MNHN-ZM-AC-1993-228 | DP2-4 M1-(2) |
|  | *Capreolus capreolus* | MNHN-ZM-AC-1993-234 | DP2-4 M1-(2) |
|  | *Capreolus capreolus* | MNHN-ZM-AC-1993-243 | DP2-4 M1-(2) |
|  | *Capreolus capreolus* | MNHN-ZM-AC-1993-229 | DP2-4 M1-2 |
|  | *Capreolus capreolus* | MNHN-ZM-AC-1993-231 | DP2-4 M1-2 |
|  | *Capreolus capreolus* | MNHN-ZM-AC-1993-198 | DP2-4 M1-2 |
|  | *Capreolus capreolus* | MNHN-ZM-AC-1958-199 | DP2-4 M1-2 |
|  | *Capreolus capreolus* | MNHN-ZM-AC-1993-239 | DP2-4 M1-2 |
|  | *Capreolus capreolus* | MNHN-ZM-AC-1993-240 | DP2-4 M1-2 |
|  | *Capreolus capreolus* | MNHN-ZM-AC-1993-253 | DP2-4 M1-2 |
|  | *Capreolus capreolus* | MNHN-ZM-AC-1993-259 | DP2-4 M1-2 |
|  | *Capreolus capreolus* | MNHN-ZM-AC-1993-261 | DP2-4 M1-2 |
|  | *Capreolus capreolus* | MNHN-ZM-AC-1993-267 | DP2-4 M1-2 |
|  | *Capreolus capreolus* | MNHN-ZM-AC-1993-268 | DP2-4 M1-2 |
|  | *Capreolus capreolus* | MNHN-ZM-AC-1993-270 | DP2-4 M1-2 |
|  | *Capreolus capreolus* | MNHN-ZM-AC-1993-281 | DP2-4 M1-2 |
|  | *Capreolus capreolus* | MNHN-ZM-AC-1927-87 | DP2-4 M1-(3) |
|  | *Capreolus capreolus* | MNHN-ZM-AC-1993-287 | DP2-4 M1-(3) |
|  | *Capreolus capreolus* | MNHN-ZM-AC-1993-141 | DP2-P3-4 M1-3 |
|  | *Capreolus capreolus* | MNHN-ZM-AC-1992-2034 | DP2-P3-4 M1-3 |
|  | *Capreolus capreolus* | MNHN-ZM-AC-1993-150 | P2-4 M1-3 |
|  | *Capreolus capreolus* | MNHN-ZM-AC-1993-151 | P2-4 M1-3 |
|  | *Capreolus capreolus* | MNHN-ZM-AC-1993-155 | P2-4 M1-3 |
|  | *Capreolus capreolus* | MNHN-ZM-AC-1993-164 | P2-4 M1-3 |
|  | *Capreolus capreolus* | MNHN-ZM-AC-1993-169 | P2-4 M1-3 |
|  | *Capreolus capreolus* | MNHN-ZM-AC-1993-180 | P2-4 M1-3 |
|  | *Capreolus capreolus* | MNHN-ZM-AC-1993-189 | P2-4 M1-3 |
|  | *Capreolus capreolus* | MNHN-ZM-AC-1993-191 | P2-4 M1-3 |
|  | *Capreolus capreolus* | MNHN-ZM-AC-1993-196 | P2-4 M1-3 |
|  | *Capreolus capreolus* | MNHN-ZM-AC-1993-224 | P2-4 M1-3 |
|  | *Capreolus capreolus* | MNHN-ZM-AC-1993-226 | P2-4 M1-3 |
|  | *Capreolus capreolus* | MNHN-ZM-AC-1993-237 | P2-4 M1-3 |
|  | *Capreolus capreolus* | MNHN-ZM-AC-1993-249 | P2-4 M1-3 |
|  | *Capreolus capreolus* | MNHN-ZM-AC-1993-251 | P2-4 M1-3 |
|  | *Capreolus capreolus* | MNHN-ZM-AC-1993-284 | P2-4 M1-3 |
|  | *Capreolus capreolus* | MNHN-ZM-AC-1993-304 | P2-4 M1-3 |
|  | *Capreolus capreolus* | MNHN-ZM-AC-1993-197 | P2-4 M1-3 |
|  | *Capreolus capreolus* | MNHN-ZM-AC-1993-202 | P2-4 M1-3 |
|  | *Capreolus capreolus* | MNHN-ZM-AC-1993-211 | P2-4 M1-3 |
|  | *Capreolus capreolus* | MNHN-ZM-AC-1993-220 | P2-4 M1-3 |
|  | *Capreolus capreolus* | MNHN-ZM-AC-1993-248 | P2-4 M1-3w |
| Bovidae | *Cervus elaphus* | MNHN-ZM-AC-1906-336 | DP2-4 M1 |
|  | *Cervus elaphus* | MNHN-ZM-MO-1993-1667 | DP2-4 M1 |
|  | *Cervus elaphus* | MNHN-ZM-MO-1933-213 | DP2-4 M1 |
|  | *Cervus elaphus* | MNHN-ZM-2013-1053 | DP2-4 M1 |
|  | *Cervus elaphus* | MNHN-ZM-2013-1043 | DP2-4 M1 |
|  | *Cervus elaphus* | MNHN-ZM-2013-1038 | DP2-4 M1 |
|  | *Cervus elaphus* | MNHN-ZM-AC-1927-54 | DP2-4 M1-(2) |
|  | *Cervus elaphus* | MNHN-ZM-AC-1933-219 | DP2-4 M1-(2) |
|  | *Cervus elaphus* | MNHN-ZM-AC-1902-923 | DP2-4 M1-2 |
|  | *Cervus elaphus* | MNHN-ZM-AC-1900-314 | DP2-4 M1-2 |
|  | *Cervus elaphus* | MNHN-ZM-AC-1951-260 | DP2-4 M1-2 |
|  | *Cervus elaphus* | MNHN-ZM-AC-2013-1050 | DP2-4 M1-2 |
|  | *Cervus elaphus* | MNHN-ZM-AC-1884-552 | P(2-4)-M1-(3) |
|  | *Cervus elaphus* | MNHN-ZM-2013-1022 | P(2)-4 M1-3 |
|  | *Cervus elaphus* | MNHN-ZM-AC-1940-159 | P2-4 M1-3 |
|  | *Cervus elaphus* | MNHN-ZM-AC-1940-429 | P2-4 M1-3 |
|  | *Cervus elaphus* | MNHN-ZM-AC-1847-453 | P2-4 M1-3 |
|  | *Cervus elaphus* | MNHN-ZM-AC-1962-1165 | P2-4 M1-3 |
|  | *Cervus elaphus* | MNHN-ZM-MO-2004-290 | P2-4 M1-3 |
|  | *Cervus elaphus* | MNHN-ZM-2013-1033 | P2-4 M1-3 |
|  | *Cervus elaphus* | MNHN-ZM-2013-1020 | P2-4 M1-3 |
|  | *Cervus elaphus* | MNHN-ZM-AC-1927-90 | P2-4 M1-3 |
|  | *Cervus elaphus* | MNHN-ZM-AC-1890-1032 | P2-4 M1-3 |
|  | *Cervus elaphus* | MNHN-ZM-2013-1048 | P2-4 M1-3 |
|  | *Cervus elaphus* | MNHN-ZM-2013-1046 | P2-4 M1-3 |
|  | *Cervus elaphus* | MNHN-ZM-AC-1938-45 | P2-4 M1-3w |
|  | *Cervus elaphus* | MNHN-ZM-AC-A12755 | P2-4 M1-3w |
|  | *Cervus elaphus* | MNHN-ZM-2013-1036 | P2-4 M1-3w |
| Hippopotamidae | *Choeropsis liberiensis* | MNHN-ZM-2007-1430 | DP2-4 M1 |
|  | *Choeropsis liberiensis* | MNHN-ZM-2017-1196 | P2-3-DP4 M1-2 |
|  | *Choeropsis liberiensis* | MNHN-ZM-AC-1948-1 | P2-4-M1-2 |
|  | *Choeropsis liberiensis* | MNHN-ZM-AC-1936-404 | P2-4-M1-(3) |
|  | *Choeropsis liberiensis* | MNHN-ZM-AC-1934-655 | P2-4 M1-3 |
|  | *Choeropsis liberiensis* | MNHN-ZM-AC-1921-309 | P2-4 M1-3 |
|  | *Choeropsis liberiensis* | MNHN-ZM-2017-1195 | P2-4 M1-3 |
|  | *Choeropsis liberiensis* | MNHN-ZM-AC-1963-104 | P2-4 M1-3w |
|  | *Choeropsis liberiensis* | MNHN-ZM-2011-897 | P2-4 M1-3w |
|  | *Choeropsis liberiensis* | MNHN-ZM-2011-896 | P2-4 M1-3w |
|  | *Choeropsis liberiensis* | MNHN-ZM-AC-1978-104 | P2-4 M1-3w |
|  | *Choeropsis liberiensis* | MNHN-ZM-AC-1982-10 | P2-4 M1-3w |
|  | *Choeropsis liberiensis* | MNHN-ZM-AC-1944-146 | P2-4 M1-3w |
| Rhinocerotidae | *Diceros bicornis* | MNHN-ZM-AC-1941-208 (L) | DP2-4 M1 |
|  | *Diceros bicornis* | MNHN-ZM-MO-1965-1126 | DP2-4 M1-2 |
|  | *Diceros bicornis* | MNHN-ZM-MO-1965-1128 | DP2-4 M1-2 |
|  | *Diceros bicornis* | MNHN-ZM-AC-1931-581 | P2-4-M1-(3) |
|  | *Diceros bicornis* | MNHN-ZM-AC-1974-124 | P2-4-M1-(3) |
|  | *Diceros bicornis* | MNHN-ZM-AC-1944-278 | P2-4 M1-3 |
|  | *Diceros bicornis* | MNHN-ZM-AC-1996-2520 | P2-4 M1-3 |
|  | *Diceros bicornis* | MNHN-ZM-AC-1961-195 | P2-4 M1-3 |
|  | *Diceros bicornis* | MNHN-ZM-MO-1965-1129 | P2-4 M1-3w |
| Equidae | *Equus burchelli* | MNHN-ZM-MO-1977-71 | DP2-4 M1 |
|  | *Equus burchelli* | MNHN-ZM-MO-1977-87 | DP2-4 M1 |
|  | *Equus burchelli* | MNHN-ZM-AC-A9843 | DP2-4 M1 |
|  | *Equus burchelli* | MNHN-ZM-2009-397 | DP2-4 M1-(2) |
|  | *Equus burchelli* | MNHN-ZM-AC-1847-340 | P2-4 M1-(3) |
|  | *Equus burchelli* | MNHN-ZM-AC-1972-07 | P2-4 M1-3 |
|  | *Equus burchelli* | MNHN-ZM-AC-1967-264 | P2-4 M1-3 |
|  | *Equus burchelli* | MNHN-ZM-AC-1932-382 | P2-4 M1-3 |
|  | *Equus burchelli* | MNHN-ZM-AC-1972-08 | P2-4 M1-3 |
|  | *Equus burchelli* | MNHN-ZM-AC-1972-09 | P2-4 M1-3 |
|  | *Equus burchelli* | MNHN-ZM-AC-1972-10 | P2-4 M1-3 |
|  | *Equus burchelli* | MNHN-ZM-MO-1977-69 | P2-4 M1-3 |
|  | *Equus burchelli* | MNHN-ZM-MO-1977-85 | P2-4 M1-3 |
|  | *Equus burchelli* | MNHN-ZM-MO-1977-74 | P2-4 M1-3 |
|  | *Equus burchelli* | MNHN-ZM-MO-1977-70 | P2-4 M1-3 |
|  | *Equus burchelli* | MNHN-ZM-AC-1979-90 | P2-4 M1-3 |
|  | *Equus burchelli* | MNHN-ZM-2009-396 | P2-4 M1-3 |
|  | *Equus burchelli* | MNHN-ZM-AC-1918-26 | P2-4 M1-3 |
|  | *Equus burchelli* | MNHN-ZM-AC-1894-570 | P2-4 M1-3 |
|  | *Equus burchelli* | MNHN-ZM-AC-1906-68 | P2-4 M1-3 |
| Equidae | *Equus caballus* | MNHN-ZM-AC-1997-7 | DP2-4 M1 |
|  | *Equus caballus* | MNHN-ZM-AC-1977-86 | DP2-4 M1 |
|  | *Equus caballus* | MNHN-ZM-2009-399 | DP2-4 M1 |
|  | *Equus caballus* | MNHN-ZM-AC-1880-232-4 | DP2-4 M1-(2) |
|  | *Equus caballus* | MNHN-ZM-AC-1880-235 | P2-(3)-DP4-M1-2 |
|  | *Equus caballus* | MNHN-ZM-AC-1926-124 | P2-(3)-DP4 M1-(3) |
|  | *Equus caballus* | MNHN-ZM-AC-A2318 | P2-(4) M1-(3) |
|  | *Equus caballus* | MNHN-ZM-AC-1971-324 | P2-(4) M1-(3) |
|  | *Equus caballus* | MNHN-ZM-AC-1985-1885 | P2-4 M1-3 |
|  | *Equus caballus* | MNHN-ZM-2005-716 | P2-4 M1-3 |
|  | *Equus caballus* | MNHN-ZM-AC-1891-107 | P2-4 M1-3 |
|  | *Equus caballus* | MNHN-ZM-AC-1880-232-1 | P2-4 M1-3 |
|  | *Equus caballus* | MNHN-ZM-AC-1880-232-2 | P2-4 M1-3 |
|  | *Equus caballus* | MNHN-ZM-AC-1880-232-3 | P2-4 M1-3 |
|  | *Equus caballus* | MNHN-ZM-AC-1880-232-7 | P2-4 M1-3 |
|  | *Equus caballus* | MNHN-ZM-AC-1880-232-5 | P2-4 M1-3 |
|  | *Equus caballus* | MNHN-ZM-AC-1880-747 | P2-4 M1-3 |
|  | *Equus caballus* | MNHN-ZM-AC-1902-822 | P2-4 M1-3 |
|  | *Equus caballus* | MNHN-ZM-AC-1901-64 | P2-4 M1-3 |
|  | *Equus caballus* | MNHN-ZM-AC-1880-743 | P2-4 M1-3 |
|  | *Equus caballus* | MNHN-ZM-AC-1976-43 | P2-4 M1-3 |
|  | *Equus caballus* | MNHN-ZM-MO-2000-358 | P2-4 M1-3w |
|  | *Equus caballus* | MNHN-ZM-2009-398 | P2-4 M1-3w |
| Procaviidae | *Heterohyrax brucei* | MNHN-ZM-MO-1893-415 | DP2-4 M1 |
|  | *Heterohyrax brucei* | MNHN-ZM-MO-1960-3613 | DP2-4 M1-(2) |
|  | *Heterohyrax brucei* | MNHN-ZM-MO-1933-2322 | DP2-4 M1-(2) |
|  | *Heterohyrax brucei* | MNHN-ZM-MO-1891-914 | DP2-4 M1-2 |
|  | *Heterohyrax brucei* | MNHN-ZM-MO-1977-18 | P2-(4) M1-2 |
|  | *Heterohyrax brucei* | MNHN-ZM-AC-1934-91 | P2-4 M1-2 |
|  | *Heterohyrax brucei* | MNHN-ZM-AC-1934-92 | P2-4 M1-2 |
|  | *Heterohyrax brucei* | MNHN-ZM-AC-1934-94 | P2-4 M1-2 |
|  | *Heterohyrax brucei* | MNHN-ZM-MO-1972-427 | P2-4 M1-2 |
|  | *Heterohyrax brucei* | MNHN-ZM-MO-1902-534 | P2-4 M1-2 |
|  | *Heterohyrax brucei* | MNHN-ZM-MO-1960-3612 | P2-4 M1-2 |
|  | *Heterohyrax brucei* | MNHN-ZM-MO-1897-655 | P2-4 M1-3 |
|  | *Heterohyrax brucei* | MNHN-ZM-MO-1902-535 | P2-4 M1-3 |
|  | *Heterohyrax brucei* | MNHN-ZM-MO-1911-578 | P2-4 M1-3 |
|  | *Heterohyrax brucei* | MNHN-ZM-MO-1972-404 | P2-4 M1-3 |
|  | *Heterohyrax brucei* | MNHN-ZM-MO-1972-405 | P2-4 M1-3 |
|  | *Heterohyrax brucei* | MNHN-ZM-MO-1972-406 | P2-4 M1-3 |
|  | *Heterohyrax brucei* | MNHN-ZM-MO-1972-407 | P2-4 M1-3 |
|  | *Heterohyrax brucei* | MNHN-ZM-MO-1974-181 | P2-4 M1-3 |
|  | *Heterohyrax brucei* | MNHN-ZM-MO-1972-424 | P2-4 M1-3w |
|  | *Heterohyrax brucei* | MNHN-ZM-MO-1972-425 | P2-4 M1-3w |
|  | *Heterohyrax brucei* | MNHN-ZM-MO-1972-426 | P2-4 M1-3w |
|  | *Heterohyrax brucei* | MNHN-ZM-MO-1972-428 | P2-4 M1-3w |
|  | *Heterohyrax brucei* | MNHN-ZM-MO-1977-273 | P2-4 M1-3w |
| Hippopotamidae | *Hippopotamus amphibius* | MNHN-ZM-AC-1925-385 | DP2-4 M1 |
|  | *Hippopotamus amphibius* | MNHN-ZM-2017-1179 | DP2-4 M1 |
|  | *Hippopotamus amphibius* | MNHN-ZM-MO-1987-163 (L) | P(2)-DP3-4 M1 |
|  | *Hippopotamus amphibius* | MNHN-ZM-AC-A2188 | P2-DP3-4 M1 |
|  | *Hippopotamus amphibius* | MNHN-ZM-AC-1988-13 | P2-(3)-DP4 M1-(2) |
|  | *Hippopotamus amphibius* | MNHN-ZM-2017-1172 | P2-DP3-4 M1-(2) |
|  | *Hippopotamus amphibius* | MNHN-ZM-AC-1926-305 | P(2-3)-DP4 M1-2 |
|  | *Hippopotamus amphibius* | MNHN-ZM-AC-1919-14 | P(2-3)-DP4 M1-2 |
|  | *Hippopotamus amphibius* | MNHN-ZM-2017-1177 | P2-3-DP4 M1-2 |
|  | *Hippopotamus amphibius* | MNHN-ZM-2017-1174 | P2-3-DP4 M1-2 |
|  | *Hippopotamus amphibius* | MNHN-ZM-2017-1191 | P2-3-DP4 M1-2 |
|  | *Hippopotamus amphibius* | MNHN-ZM-AC-1911-351 | P2-3-DP4 M1-(3) |
|  | *Hippopotamus amphibius* | MNHN-ZM-MO-1965-1082 | P2-(4) M1-(3) |
|  | *Hippopotamus amphibius* | MNHN-ZM-AC-1885-672 | P2-4 M1-(3) |
|  | *Hippopotamus amphibius* | MNHN-ZM-MO-1895-468 | P2-4 M1-(3) |
|  | *Hippopotamus amphibius* | MNHN-ZM-MO-2001-2118 | P2-4 M1-3 |
|  | *Hippopotamus amphibius* | MNHN-ZM-MO-1965-1080 | P2-4 M1-3 |
|  | *Hippopotamus amphibius* | MNHN-ZM-AC-1959-131 | P2-4 M1-3 |
|  | *Hippopotamus amphibius* | MNHN-ZM-AC-1917-249 | P2-4 M1-3 |
|  | *Hippopotamus amphibius* | MNHN-ZM-MO-1985-168 | P2-4 M1-3 |
|  | *Hippopotamus amphibius* | MNHN-ZM-AC-1930-217 | P2-4 M1-3 |
|  | *Hippopotamus amphibius* | MNHN-ZM-MO-1985-179 | P2-4 M1-3w |
|  | *Hippopotamus amphibius* | MNHN-ZM-2017-1188 | P2-4 M1-3w |
|  | *Hippopotamus amphibius* | MNHN-ZM-MO-2001-2117 | P2-4 M1-3w |
|  | *Hippopotamus amphibius* | MNHN-ZM-MO-1965-1081 | P2-4 M1-3w |
|  | *Hippopotamus amphibius* | MNHN-ZM-MO-1985-172 | P2-4 M1-3w |
|  | *Hippopotamus amphibius* | MNHN-ZM-AC-1926-329 | P2-4 M1-3w |
|  | *Hippopotamus amphibius* | MNHN-ZM-AC-1885-671 | P2-4 M1-3w |
|  | *Hippopotamus amphibius* | MNHN-ZM-AC-1924-138 | P2-4 M1-3w |
|  | *Hippopotamus amphibius* | MNHN-ZM-AC-1896-10 | P2-4 M1-3w |
| Bovidae | *Kobus kob* | MNHN-ZM-MO-1977-49 | DP2-4 M1 |
|  | *Kobus kob* | MNHN-ZM-MO-1985-393 | DP2-4 M1 |
|  | *Kobus kob* | MNHN-ZM-AC-1963-120 | DP2-4 M1 |
|  | *Kobus kob* | MNHN-ZM SSN8 | DP2-4 M1 |
|  | *Kobus kob* | MNHN-ZM SSN4 | DP2-4 M1-2 |
|  | *Kobus kob* | MNHN-ZM SSN5 | DP2-4 M1-2 |
|  | *Kobus kob* | MNHN-ZM-AC-1972-54 | DP2-4 M1-2 |
|  | *Kobus kob* | MNHN-ZM-AC-1936-456 | DP2-4 M1-2 |
|  | *Kobus kob* | MNHN-ZM SSN1 (1892) | DP2-4 M1-(3) |
|  | *Kobus kob* | MNHN-ZM SSN2 (119) | P2-4 M1-3 |
|  | *Kobus kob* | MNHN-ZM-MO-1965-1086 | P2-4 M1-3 |
|  | *Kobus kob* | MNHN-ZM-MO-1970-54 | P2-4 M1-3 |
|  | *Kobus kob* | MNHN-ZM SSN7 | P2-4 M1-3 |
|  | *Kobus kob* | MNHN-ZM-MO-1905-406 | P2-4 M1-3 |
|  | *Kobus kob* | MNHN-ZM-AC-1945-117 | P2-4 M1-3 |
|  | *Kobus kob* | MNHN-ZM-AC-1945-116 | P2-4 M1-3 |
|  | *Kobus kob* | MNHN-ZM-AC-1937-366 | P2-4 M1-3 |
|  | *Kobus kob* | MNHN-ZM SSN6 | P2-4 M1-3w |
| Moschidae | *Moschus moschiferans* | MNHN-ZM-MO-1874-682 | DP2-4 M1-(2) |
|  | *Moschus moschiferans* | MNHN-ZM-MO-1892-836 | DP2-4 M1-2 |
|  | *Moschus moschiferans* | MNHN-ZM-MO-1971-35 | P2-4 M1-3 |
|  | *Moschus moschiferans* | MNHN-ZM-MO-1892-843 | P2-4 M1-3 |
|  | *Moschus moschiferans* | MNHN-ZM-MO-1874-730 | P2-4 M1-3 |
|  | *Moschus moschiferans* | MNHN-ZM-MO-1891-414 | P2-4 M1-3 |
|  | *Moschus moschiferans* | MNHN-ZM-MO-1892-845 | P2-4 M1-3 |
|  | *Moschus moschiferans* | MNHN-ZM SSN2 | P2-4 M1-3 |
|  | *Moschus moschiferans* | MNHN-ZM-AC-1961-294 | P2-4 M1-3 |
|  | *Moschus moschiferans* | MNHN-ZM-MO-1962-4183 | P2-4 M1-3 |
|  | *Moschus moschiferans* | MNHN-ZM-2006-505 | P2-4 M1-3 |
|  | *Moschus moschiferans* | MNHN-ZM SSN1 | P2-4 M1-3w |
|  | *Moschus moschiferans* | MNHN-ZM SSN3 | P2-4 M1-3w |
| Bovidae | *Nanger soemmeringi* | MNHN-ZM-MO-1911-2129 | DP2-4 M1-(2) |
|  | *Nanger soemmeringi* | MNHN-ZM-MO-1965-398 | DP2-4 M1-(2) |
|  | *Nanger soemmeringi* | MNHN-ZM-MO-1972-444 | DP2-4 M1-2 |
|  | *Nanger soemmeringi* | MNHN-ZM-AC-1930-222 | DP2-4 M1-2 |
|  | *Nanger soemmeringi* | MNHN-ZM-MO-1972-443 | DP2-4 M1-2 |
|  | *Nanger soemmeringi* | MNHN-ZM-MO-1972-447 | DP2-4 M1-3 |
|  | *Nanger soemmeringi* | MNHN-ZM-MO-1972-446 | DP2-P(3-4) M1-3 |
|  | *Nanger soemmeringi* | MNHN-ZM SSN2 (4) | P(2-4) M1-3 |
|  | *Nanger soemmeringi* | MNHN-ZM-MO-1908-204 | P2-4 M1-3 |
|  | *Nanger soemmeringi* | MNHN-ZM-MO-1972-448 | P2-4 M1-3 |
|  | *Nanger soemmeringi* | MNHN-ZM-AC-1882-3 | P2-4 M1-3 |
|  | *Nanger soemmeringi* | MNHN-ZM-MO-1937-732 | P2-4 M1-3 |
|  | *Nanger soemmeringi* | MNHN-ZM SSN1 (5) | P2-4 M1-3 |
|  | *Nanger soemmeringi* | MNHN-ZM SSN3 (3) | P2-4 M1-3 |
|  | *Nanger soemmeringi* | MNHN-ZM-AC-1964-247 | P2-4 M1-3 |
|  | *Nanger soemmeringi* | MNHN-ZM-AC-1963-253 | P2-4 M1-3 |
|  | *Nanger soemmeringi* | MNHN-ZM-MO-1972-445 | P2-4 M1-3 |
|  | *Nanger soemmeringi* | MNHN-ZM-MO-1908-204A | P2-4 M1-3 |
|  | *Nanger soemmeringi* | MNHN-ZM-MO-1911-2127 | P2-4 M1-3w |
|  | *Nanger soemmeringi* | MNHN-ZM-MO-1911-2128 | P2-4 M1-3w |
|  | *Nanger soemmeringi* | MNHN-ZM-MO-1940-1154 | P2-4 M1-3w |
| Bovidae | *Ourebia ourebi* | MNHN-ZM-MO-1904-2032 | DP2-4 M1 |
|  | *Ourebia ourebi* | MNHN-ZM-MO-1970-48 | DP2-4 M1 |
|  | *Ourebia ourebi* | MNHN-ZM-MO-1961-171 | DP2-4 M1 |
|  | *Ourebia ourebi* | MNHN-ZM-MO-1911-2328 | DP2-4 M1-(2) |
|  | *Ourebia ourebi* | MNHN-ZM-MO-1904-2037 | DP2-4 M1-2 |
|  | *Ourebia ourebi* | MNHN-ZM-MO-A11041 | DP2-4 M1-2 |
|  | *Ourebia ourebi* | MNHN-ZM-2007-1394 | DP2-4 M1-2 |
|  | *Ourebia ourebi* | MNHN-ZM-MO-1962-1003 | P(2-4) M1-2 |
|  | *Ourebia ourebi* | MNHN-ZM-AC-1921-235 | P(2-4) M1-2 |
|  | *Ourebia ourebi* | MNHN-ZM SSN6 (54) | P2-4 M1-(3) |
|  | *Ourebia ourebi* | MNHN-ZM-2007-1395 | P2-4 M1-(3) |
|  | *Ourebia ourebi* | MNHN-ZM SSN1 (6) | P2-4 M1-3 |
|  | *Ourebia ourebi* | MNHN-ZM-MO-1908-199 | P2-4 M1-3 |
|  | *Ourebia ourebi* | MNHN-ZM-MO-1970-47 | P2-4 M1-3 |
|  | *Ourebia ourebi* | MNHN-ZM-MO-AE691 | P2-4 M1-3 |
|  | *Ourebia ourebi* | MNHN-ZM SSN5 (3) | P2-4 M1-3 |
|  | *Ourebia ourebi* | MNHN-ZM-MO-1968-778 | P2-4 M1-3 |
|  | *Ourebia ourebi* | MNHN-ZM-MO-1908-199-2 | P2-4 M1-3 |
|  | *Ourebia ourebi* | MNHN-ZM-2007-1393 | P2-4 M1-3 |
|  | *Ourebia ourebi* | MNHN-ZM-MO-1970-49 | P2-4 M1-3 |
|  | *Ourebia ourebi* | MNHN-ZM-MO-1908-200 | P2-4 M1-3 |
|  | *Ourebia ourebi* | MNHN-ZM SSN2 (5) | P2-4 M1-3w |
|  | *Ourebia ourebi* | MNHN-ZM SSN3 | P2-4 M1-3w |
|  | *Ourebia ourebi* | MNHN-ZM SSN4 | P2-4 M1-3w |
|  | *Ourebia ourebi* | MNHN-ZM-MO-1971-18 | P2-4 M1-3w |
|  | *Ourebia ourebi* | MNHN-ZM-MO-1970-45 | P2-4 M1-3w |
| Tayassuidae | *Pecari tajacu* | MNHN-ZM-AC-1994-95 | DP2-4 M1 |
|  | *Pecari tajacu* | MNHN-ZM-AC-1880-615 | DP2-4 M1-2 |
|  | *Pecari tajacu* | MNHN-ZM SSN1 | DP2-4 M1-2 |
|  | *Pecari tajacu* | MNHN-ZM-AC-1902-1426 | DP2-P(3-4) M1-2 |
|  | *Pecari tajacu* | MNHN-ZM-AC-1884-557 | P2-4 M1-(3) |
|  | *Pecari tajacu* | MNHN-ZM-AC-1884-574 | P2-4 M1-(3) |
|  | *Pecari tajacu* | MNHN-ZM-MO-1937-733 | P2-4 M1-3 |
|  | *Pecari tajacu* | MNHN-ZM-AC-1971-78 | P2-4 M1-3 |
|  | *Pecari tajacu* | MNHN-ZM-AC-1968-785 | P2-4 M1-3 |
|  | *Pecari tajacu* | MNHN-ZM-AC-1901-634 | P2-4 M1-3 |
|  | *Pecari tajacu* | MNHN-ZM-AC-1870-132 | P2-4 M1-3 |
|  | *Pecari tajacu* | MNHN-ZM-AC-1967-942 | P2-4 M1-3 |
|  | *Pecari tajacu* | MNHN-ZM-AC-1968-786 | P2-4 M1-3 |
|  | *Pecari tajacu* | MNHN-ZM-MO-1981-439 | P2-4 M1-3 |
|  | *Pecari tajacu* | MNHN-ZM-AC-1917-263 | P2-4 M1-3 |
|  | *Pecari tajacu* | MNHN-ZM-AC-1870-596 | P2-4 M1-3 |
|  | *Pecari tajacu* | MNHN-ZM-AC-1927-2365 | P2-4 M1-3 |
|  | *Pecari tajacu* | MNHN-ZM-AC-A13390 | P2-4 M1-3 |
|  | *Pecari tajacu* | MNHN-ZM-AC-1902-942 | P2-4 M1-3 |
|  | *Pecari tajacu* | MNHN-ZM-AC-1879-192 | P2-4 M1-3 |
|  | *Pecari tajacu* | MNHN-ZM-AC-A2173 | P2-4 M1-3 |
|  | *Pecari tajacu* | MNHN-ZM-AC-2000-352 | P2-4 M1-3w |
| Suidae | *Potamochoerus porcus* | MNHN-ZM-AC-1903-129 | DP2-4 M1 |
|  | *Potamochoerus porcus* | MNHN-ZM-AC-1950-234 | DP2-4 M1 |
|  | *Potamochoerus porcus* | MNHN-ZM-AC-1927-313 | P(2-3)-DP4 M1-(2) |
|  | *Potamochoerus porcus* | MNHN-ZM-MO-1916-96 | P2-4 M1-2 |
|  | *Potamochoerus porcus* | MNHN-ZM-2013-1307 | P2-4 M1-(3) |
|  | *Potamochoerus porcus* | MNHN-ZM-AC-1928-1911 | P2-4 M1-(3) |
|  | *Potamochoerus porcus* | MNHN-ZM-AC-1904-281 | P2-4 M1-(3) |
|  | *Potamochoerus porcus* | MNHN-ZM-AC-1927-291 | P2-4 M1-(3) |
|  | *Potamochoerus porcus* | MNHN-ZM-2007-1467 | P2-4 M1-(3) |
|  | *Potamochoerus porcus* | MNHN-ZM-AC-1964-25 | P2-4 M1-3 |
|  | *Potamochoerus porcus* | MNHN-ZM-2013-1308 | P2-4 M1-3 |
|  | *Potamochoerus porcus* | MNHN-ZM-MO-2001-2128 | P2-4 M1-3 |
|  | *Potamochoerus porcus* | MNHN-ZM-AC-1855-19 | P2-4 M1-3 |
|  | *Potamochoerus porcus* | MNHN-ZM-MO-1962-1136 | P2-4 M1-3 |
|  | *Potamochoerus porcus* | MNHN-ZM-2013-1304 | P2-4 M1-3 |
|  | *Potamochoerus porcus* | MNHN-ZM-2013-1303 | P2-4 M1-3 |
|  | *Potamochoerus porcus* | MNHN-ZM-AC-1938-816 | P2-4 M1-3w |
|  | *Potamochoerus porcus* | MNHN-ZM-AC-1938-817 | P2-4 M1-3w |
|  | *Potamochoerus porcus* | MNHN-ZM-AC-1938-818 | P2-4 M1-3w |
|  | *Potamochoerus porcus* | MNHN-ZM-2013-1301 | P2-4 M1-3w |
|  | *Potamochoerus porcus* | MNHN-ZM-2013-1305 | P2-4 M1-3w |
|  | *Potamochoerus porcus* | MNHN-ZM-AC-1996-2158 | P2-4 M1-3w |
| Procaviidae | *Procavia capensis* | MNHN-ZM-MO-1969-483 | DP2-4 M1 |
|  | *Procavia capensis* | MNHN-ZM-MO-1969-484 | DP2-4 M1 |
|  | *Procavia capensis* | MNHN-ZM-MO-1977-284 | DP2-4 M1 |
|  | *Procavia capensis* | MNHN-ZM-MO-1905-387 | P(1-3)-DP4 M1 |
|  | *Procavia capensis* | MNHN-ZM-2007-391 | P1-3-DP4 M1-(2) |
|  | *Procavia capensis* | MNHN-ZM-MO-1977-302 | P1-4 M1-(2) |
|  | *Procavia capensis* | MNHN-ZM-MO-1969-486 | P1-4 M1-2 |
|  | *Procavia capensis* | MNHN-ZM-MO-1977-310 | P1-4 M1-2 |
|  | *Procavia capensis* | MNHN-ZM-MO-1977-279 | P1-4 M1-2 |
|  | *Procavia capensis* | MNHN-ZM-MO-1977-280 | P1-4 M1-2 |
|  | *Procavia capensis* | MNHN-ZM-MO-1977-301 | P1-4 M1-2 |
|  | *Procavia capensis* | MNHN-ZM-MO-1911-832 | P1-4 M1-2 |
|  | *Procavia capensis* | MNHN-ZM-MO-1962-846 | P1-4 M1-2 |
|  | *Procavia capensis* | MNHN-ZM-MO-1911-833 | P2-4 M1-(3) |
|  | *Procavia capensis* | MNHN-ZM-MO-1972-414 | P2-4 M1-(3) |
|  | *Procavia capensis* | MNHN-ZM-MO-1977-282 | P2-4 M1-(3) |
|  | *Procavia capensis* | MNHN-ZM-MO-1972-412 | P2-4 M1-(3) |
|  | *Procavia capensis* | MNHN-ZM-MO-1911-1033 | P2-4 M1-3 |
|  | *Procavia capensis* | MNHN-ZM-MO-1972-410 | P2-4 M1-3 |
|  | *Procavia capensis* | MNHN-ZM-MO-1972-418 | P2-4 M1-3 |
|  | *Procavia capensis* | MNHN-ZM-MO-2001-1989 | P2-4 M1-3 |
|  | *Procavia capensis* | MNHN-ZM-MO-2001-1992 | P2-4 M1-3 |
|  | *Procavia capensis* | MNHN-ZM-AC-1979-39 | P2-4 M1-3 |
|  | *Procavia capensis* | MNHN-ZM-MO-1891-912 | P2-4 M1-3 |
|  | *Procavia capensis* | MNHN-ZM-MO-1972-416 | P2-4 M1-3 |
|  | *Procavia capensis* | MNHN-ZM-2007-393 | P2-4 M1-3 |
|  | *Procavia capensis* | MNHN-ZM-MO-1951-1056 | P2-4 M1-3w |
|  | *Procavia capensis* | MNHN-ZM-MO-1972-423 | P2-4 M1-3w |
|  | *Procavia capensis* | MNHN-ZM-2007-395 | P2-4 M1-3w |
|  | *Procavia capensis* | MNHN-ZM-MO-1972-431 | P2-4 M1-3w |
| Bovidae | *Rupicapra pyrenaica* | MNHN-ZM-2007-400 | DP2-4 M1 |
|  | *Rupicapra pyrenaica* | MNHN-ZM-2007-401 | DP2-4 M1 |
|  | *Rupicapra pyrenaica* | MNHN-ZM-2007-402 | DP2-4 M1 |
|  | *Rupicapra rupicapra* | MNHN-ZM-2009-418 | DP2-4 M1-(2) |
|  | *Rupicapra pyrenaica* | MNHN-ZM-2007-1345 | DP2-4 M1-2 |
|  | *Rupicapra rupicapra* | MNHN-ZM-2007-1427 | DP2-4 M1-2 |
|  | *Rupicapra pyrenaica* | MNHN-ZM-2007-1346 | P(2-4)-M1-2 |
|  | *Rupicapra rupicapra* | MNHN-ZM-2007-1331 | P(2-4)-M1-(3) |
|  | *Rupicapra pyrenaica* | MNHN-ZM-2007-1339 | P2-4 M1-(3) |
|  | *Rupicapra pyrenaica* | MNHN-ZM-2007-1340 | P2-4 M1-3 |
|  | *Rupicapra pyrenaica* | MNHN-ZM-2007-1342 | P2-4 M1-3 |
|  | *Rupicapra pyrenaica* | MNHN-ZM-2007-1332 | P2-4 M1-3 |
|  | *Rupicapra pyrenaica* | MNHN-ZM-2007-1343 | P2-4 M1-3 |
|  | *Rupicapra pyrenaica* | MNHN-ZM-2007-1348 | P2-4 M1-3 |
|  | *Rupicapra rupicapra* | MNHN-ZM-2009-419 | P2-4 M1-3 |
|  | *Rupicapra rupicapra* | MNHN-ZM-2009-420 | P2-4 M1-3 |
|  | *Rupicapra rupicapra* | MNHN-ZM-2007-1333 | P2-4 M1-3 |
|  | *Rupicapra rupicapra* | MNHN-ZM-2007-1344 | P2-4 M1-3 |
|  | *Rupicapra rupicapra* | MNHN-ZM-2007-1338 | P2-4 M1-3 |
|  | *Rupicapra rupicapra* | MNHN-ZM-2007-1334 | P2-4 M1-3 |
|  | *Rupicapra rupicapra* | MNHN-ZM-2007-1341 | P2-4 M1-3 |
|  | *Rupicapra pyrenaica* | MNHN-ZM-2007-1347 | P2-4 M1-3w |
|  | *Rupicapra rupicapra* | MNHN-ZM-2009-417 | P2-4 M1-3w |
| Suidae | *Sus scrofa* | OL1H279 | DP2-4 M1 |
|  | *Sus scrofa* | OL1H282 | DP2-4 M1 |
|  | *Sus scrofa* | OL1H285 | DP2-4 M1 |
|  | *Sus scrofa* | OL1H288 | DP2-4 M1 |
|  | *Sus scrofa* | OL2H279 | DP2-4 M1 |
|  | *Sus scrofa* | OL2H282 | DP2-4 M1 |
|  | *Sus scrofa* | OL2H283 | DP2-4 M1 |
|  | *Sus scrofa* | OL2H293 | DP2-4 M1 |
|  | *Sus scrofa* | OL2H313 | DP2-4 M1 |
|  | *Sus scrofa* | OL3H279 | DP2-4 M1-(2) |
|  | *Sus scrofa* | OL3H282 | DP2-4 M1-(2) |
|  | *Sus scrofa* | OL3H292 | DP2-4 M1-(2) |
|  | *Sus scrofa* | OL3H286 | DP2-4 M1-2 |
|  | *Sus scrofa* | OL3H288 | DP2-4 M1-2 |
|  | *Sus scrofa* | OL4H282 | DP2-4 M1-2 |
|  | *Sus scrofa* | OL4H283 | DP2-4 M1-2 |
|  | *Sus scrofa* | OL4H316 | DP2-4 M1-2 |
|  | *Sus scrofa* | OL4H332 | P(2-4) M1-2 |
|  | *Sus scrofa* | OL4H288 | P(2-4) M1-2 |
|  | *Sus scrofa* | OL4H291 | P2-4 M1-2 |
|  | *Sus scrofa* | OL5H279 | P2-4 M1-2 |
|  | *Sus scrofa* | OL5H282 | P2-4 M1-2 |
|  | *Sus scrofa* | OL6H289 | P2-4 M1-2 |
|  | *Sus scrofa* | OL6H292 | P2-4 M1-2 |
|  | *Sus scrofa* | OL5H291 | P2-4 M1-(3) |
|  | *Sus scrofa* | OL5H293 | P2-4 M1-(3) |
|  | *Sus scrofa* | OL5H312 | P2-4 M1-(3) |
|  | *Sus scrofa* | OL6H279 | P2-4 M1-(3) |
|  | *Sus scrofa* | OL6H282 | P2-4 M1-(3) |
|  | *Sus scrofa* | OL6H291 | P2-4 M1-(3) |
|  | *Sus scrofa* | OL6H293 | P2-4 M1-(3) |
|  | *Sus scrofa* | OL6H312 | P2-4 M1-(3) |
|  | *Sus scrofa* | OL6H319 | P2-4 M1-(3) |
|  | *Sus scrofa* | OL6H320 | P2-4 M1-(3) |
|  | *Sus scrofa* | OL6H330 | P2-4 M1-(3) |
|  | *Sus scrofa* | OL6H331 | P2-4 M1-(3) |
|  | *Sus scrofa* | OL6H332 | P2-4 M1-(3) |
|  | *Sus scrofa* | OL6H334 | P2-4 M1-(3) |
|  | *Sus scrofa* | OL6H313 | P2-4 M1-3 |
|  | *Sus scrofa* | OL6H314 | P2-4 M1-3 |
|  | *Sus scrofa* | OL6H321 | P2-4 M1-3 |
| Tapiridae | *Tapirus terrestris* | MNHN-ZM-AC-1931-593 | DP2-4 M1 |
|  | *Tapirus terrestris* | MNHN-ZM-AC-1956-160 | DP2-4 M1 |
|  | *Tapirus terrestris* | MNHN-ZM-2016-2809 | DP2-4 M1 |
|  | *Tapirus terrestris* | MNHN-ZM-2017-15 | DP2-4 M1 |
|  | *Tapirus terrestris* | MNHN-ZM-AC-1932-319 | P2-3-DP4 M1 |
|  | *Tapirus terrestris* | MNHN-ZM-AC-1972-178 | P2-3-DP4 M1 |
|  | *Tapirus terrestris* | MNHN-ZM-AC-1889-50 | P2-3-DP4 M1 |
|  | *Tapirus terrestris* | MNHN-ZM-2017-13 | P2-3-DP4 M1-2 |
|  | *Tapirus terrestris* | MNHN-ZM-2017-19 | P2-4 M1-2 |
|  | *Tapirus terrestris* | MNHN-ZM-AC-1937-01 | P2-4 M1-2 |
|  | *Tapirus terrestris* | MNHN-ZM-MO-2001-1615 | P2-4 M1-2 |
|  | *Tapirus terrestris* | MNHN-ZM-2017-18 | P2-4 M1-2 |
|  | *Tapirus terrestris* | MNHN-ZM-MO-1985-1886 | P2-4 M1-2 |
|  | *Tapirus terrestris* | MNHN-ZM-AC-1939-225 | P2-4 M1-(3) |
|  | *Tapirus terrestris* | MNHN-ZM-AC-1928-287 | P2-4 M1-3 |
|  | *Tapirus terrestris* | MNHN-ZM-AC-1938-436 | P2-4 M1-3 |
|  | *Tapirus terrestris* | MNHN-ZM-MO-1990-20 | P2-4 M1-3 |
|  | *Tapirus terrestris* | MNHN-ZM-2017-14 | P2-4 M1-3 |
|  | *Tapirus terrestris* | MNHN-ZM-AC-A2265 | P2-4 M1-3 |
|  | *Tapirus terrestris* | MNHN-ZM-MO-1948-612 | P2-4 M1-3 |
|  | *Tapirus terrestris* | MNHN-ZM-2016-2810 | P2-4 M1-3 |
| Bovidae | *Tragelaphus scriptus* | MNHN-ZM-AC-1910-25 | DP2-4 M1 |
|  | *Tragelaphus scriptus* | MNHN-ZM-AC-1966-205 | DP2-4 M1 |
|  | *Tragelaphus scriptus* | MNHN-ZM-MO-1963-903 | DP2-4 M1 |
|  | *Tragelaphus scriptus* | MNHN-ZM-MO-1963-900 | DP2-4 M1 |
|  | *Tragelaphus scriptus* | MNHN-ZM-MO-1940-1180 | DP2-4 M1 |
|  | *Tragelaphus scriptus* | MNHN-ZM-AC-1922-57 | DP2-4 M1-(2) |
|  | *Tragelaphus scriptus* | MNHN-ZM-MO-1904-2023 | DP2-4 M1-(2) |
|  | *Tragelaphus scriptus* | MNHN-ZM-AC-1898-49 | DP2-4 M1-2 |
|  | *Tragelaphus scriptus* | MNHN-ZM-AC-1912-293 | DP2-4 M1-2 |
|  | *Tragelaphus scriptus* | MNHN-ZM-MO-1963-901 | DP2-4 M1-2 |
|  | *Tragelaphus scriptus* | MNHN-ZM-AC-1963-905 | DP2-4 M1-2 |
|  | *Tragelaphus scriptus* | MNHN-ZM-MO-1973-153 | DP2-4 M1-2 |
|  | *Tragelaphus scriptus* | MNHN-ZM-2007-1286 | DP2-4 M1-(3) |
|  | *Tragelaphus scriptus* | MNHN-ZM-MO-1939-681 | P2-(4) M1-3 |
|  | *Tragelaphus scriptus* | MNHN-ZM-MO-1905-416 | P(2-4) M1-3 |
|  | *Tragelaphus scriptus* | MNHN-ZM-2007-1285 | P2-4 M1-3 |
|  | *Tragelaphus scriptus* | MNHN-ZM-2007-1293 | P2-4 M1-3 |
|  | *Tragelaphus scriptus* | MNHN-ZM-2007-1289 | P2-4 M1-3 |
|  | *Tragelaphus scriptus* | MNHN-ZM-2007-1291 | P2-4 M1-3 |
|  | *Tragelaphus scriptus* | MNHN-ZM-MO-1969-493 | P2-4 M1-3 |
|  | *Tragelaphus scriptus* | MNHN-ZM-2007-1288 | P2-4 M1-3 |
|  | *Tragelaphus scriptus* | MNHN-ZM-MO-1966-206 | P2-4 M1-3 |
|  | *Tragelaphus scriptus* | MNHN-ZM-2007-1290 | P2-4 M1-3 |
|  | *Tragelaphus scriptus* | MNHN-ZM-2007-486 | P2-4 M1-3 |
|  | *Tragelaphus scriptus* | MNHN-ZM-AC-1921-182 | P2-4 M1-3 |
|  | *Tragelaphus scriptus* | MNHN-ZM-MO-1969-495 | P2-4 M1-3 |
|  | *Tragelaphus scriptus* | MNHN-ZM-MO-1884-2391 | P2-4 M1-3 |
|  | *Tragelaphus scriptus* | MNHN-ZM-MO-1953-859 | P2-4 M1-3 |
|  | *Tragelaphus scriptus* | MNHN-ZM-2007-1287 | P2-4 M1-3w |
|  | *Tragelaphus scriptus* | MNHN-ZM-MO-1969-494 | P2-4 M1-3w |
| Tragulidae | *Tragulus javanicus* | MNHN-ZM-AC-A11292 | DP2-4 M1 |
|  | *Tragulus* sp. | MNHN-ZM-AC-1874-189 | DP2-4 M1 |
|  | *Tragulus javanicus* | MNHN-ZM-MO-1963-1396 | DP2-4 M1-2 |
|  | *Tragulus javanicus* | MNHN-ZM-AC-A11294 | DP2-4 M1-2 |
|  | *Tragulus javanicus* | MNHN-ZM-MO-1982-860 | DP2-4 M1-(3) |
|  | *Tragulus* sp. | MNHN-ZM-2007-1415 | DP2-4 M1-(3) |
|  | *Tragulus javanicus* | MNHN-ZM-2007-1409 | DP2-4 M1-3 |
|  | *Tragulus javanicus* | MNHN-ZM-MO-1971-29 | P2-4 M1-3 |
|  | *Tragulus javanicus* | MNHN-ZM-MO-1976-392 | P2-4 M1-3 |
|  | *Tragulus javanicus* | MNHN-ZM-AC-1871-333 | P2-4 M1-3 |
|  | *Tragulus javanicus* | MNHN-ZM-AC-1964-297 | P2-4 M1-3 |
|  | *Tragulus javanicus* | MNHN-ZM-AC-A3370 | P2-4 M1-3 |
|  | *Tragulus javanicus* | MNHN-ZM-AC-A11296 | P2-4 M1-3 |
|  | *Tragulus* sp. | MNHN-ZM SSN1 | P2-4 M1-3 |
|  | *Tragulus* sp. | MNHN-ZM-AC-A12497 | P2-4 M1-3 |
|  | *Tragulus* sp. | MNHN-ZM-AC-1883-354 | P2-4 M1-3 |
|  | *Tragulus* sp. | MNHN-ZM-AC-1861-57 | P2-4 M1-3 |
|  | *Tragulus javanicus* | MNHN-ZM-AC-A12499 | P2-4 M1-3w |
|  | *Tragulus* sp. | MNHN-ZM-AC-1929-402 | P2-4 M1-3w |
|  | *Tragulus* sp. | MNHN-ZM SSN2 | P2-4 M1-3w |
