# Supplementary material for: Accommodation of Dental Variations During Jaw Growth in Ungulate Mammals
Source: J Exp Zool B Mol Dev Evol. 2025 Aug 5;344(8):487–504. doi: 10.1002/jez.b.23321 (PMC12626908; doi:10.1002/jez.b.23321)
Supplement: Supplementary file 2 — Online resource 2: List of landmarks used in this study. Mesial and labial sides of cheek teeth correspond to their anterior and posterior parts, respectively. [file JEZ-344-487-s004.docx]

**Online resource 2.** List of landmarks used in this study. Mesial and labial sides of cheek teeth correspond to their anterior and posterior parts, respectively.

| **Numbers** | **Definition** |
| --- | --- |
| 1 | Most ventro-medial point of the premaxilla-maxilla suture near the incisive foramen |
| 2 | Postero-medial point of the palatine (near the choanae) |
| 3 | Mesio-labial point of the 2nd premolar |
| 4 | Mesio-lingual point of the 3rd premolar |
| 5 | Mesio-labial point of the 3rd premolar |
| 6 | Mesio-lingual point of the 4th premolar |
| 7 | Mesio-labial point of the 4th premolar |
| 8 | Mesio-lingual point of the 1st molar |
| 9 | Mesio-labial point of the 1st molar |
| 10 | Disto-lingual point of the 1st molar |
| 11 | Disto-labial point of the 1st molar |
| 12 | Most postero-ventral point of the maxilla close to the palatine |
| 13-20 | Semi-landmarks on the labial margin of the maxilla |
| 21 | Most ventro-lateral point of the maxilla-premaxilla suture |
