# Supplementary material for: Accommodation of Dental Variations During Jaw Growth in Ungulate Mammals
Source: J Exp Zool B Mol Dev Evol. 2025 Aug 5;344(8):487–504. doi: 10.1002/jez.b.23321 (PMC12626908; doi:10.1002/jez.b.23321)
Supplement: Supplementary file 4 — Online resource 4: Ratios of palate centroid size at different stages of molar eruption. [file JEZ-344-487-s003.docx]

**Online resource 3** Ratios of palate centroid size at different stages of molar eruption. CS M1/M3: mean palate centroid size at *M1 erupted* */* mean Csize palate at *M3 erupted*; CS M2/M3: mean palate centroid size at *M2 erupted /* mean Csize palate at *M3 erupted*. The asterisk indicates ratios including only one specimen for CS M1 or CS M2.

| Order | Family | Species | CS M1/M3 | CS M2/M3 |
| --- | --- | --- | --- | --- |
| Hyracoidea | Procaviidae | *Procavia capensis* | 0.80 | 0.92 |
|  |  | *Heterohyrax brucei* | 0.78* | 0.94 |
| Perissodactyla | Tapiridae | *Tapirus terrestris* | 0.89 | 1.01 |
|  | Equidae | *Equus caballus* | 0.78 | 1.01 |
|  |  | *Equus burchelli* | 0.87 | - |
|  | Rhinocerotidae | *Diceros bicornis* | 0.74* | 0.96 |
| Artiodactyla | Hippopotamidae | *Hippopotamus amphibius* | 0.72 | 0.86 |
|  |  | *Choeropsis liberiensis* | 0.68 | 0.96 |
|  | Suidae | *Sus scrofa* | 0.70 | 0.91 |
|  |  | *Potamochoerus porcus* | 0.59 | 0.83* |
|  | Tayassuidae | *Pecari tajacu* | 0.86* | 0.96 |
|  | Tragulidae | *Tragulus spp.* | 0.74 | 0.94 |
|  | Cervidae | *Capreolus capreolus* | 0.82 | 0.90 |
|  |  | *Cervus elaphus* | 0.77 | 0.88 |
|  | Moschidae | *Moschus moschiferus* | - | 0.80* |
|  | Bovidae | *Ammotragus lervia* | 0.77 | 1.02 |
|  |  | *Rupicapra spp.* | 0.80 | 0.98 |
|  |  | *Ourebia ourebi* | 0.85 | 1.01 |
|  |  | *Nanger soemmerringii* | - | 1.03 |
|  |  | *Tragelaphus scriptus* | 0.84 | 0.93 |
|  |  | *Alcelaphus buselaphus* | 0.82* | 1.03* |
|  |  | *Capra hircus* | 0.76 | 0.94 |
|  |  | *Kobus kob* | 0.80 | 0.96 |
